# Heavy metal ion detection using green precursor derived carbon dots

**DOI:** 10.1016/j.isci.2022.103816

**Published:** 2022-01-25

**Authors:** Simei Darinel Torres Landa, Naveen Kumar Reddy Bogireddy, Inderbir Kaur, Vandana Batra, Vivechana Agarwal

**Affiliations:** 1 Center for Research Engineering and Applied Sciences, Autonomous State University of Morelos (CIICAp-UAEM), Av. Univ. 1001, Col. Chamilpa, Cuernavaca, Morelos 62209, Mexico; 2 Physics Institute, National Autonomous University of Mexico (IF-UNAM), Distrito Federal C.P. 04510, México; 3 Department of Electronics, Bhaskaracharya College of Applied Sciences, University of Delhi, Delhi 110075, India; 4 Department of Physics, Bhaskaracharya College of Applied Sciences, University of Delhi, Delhi 110075, India

**Keywords:** Chemistry, Green chemistry, Materials science

## Abstract

The discovery of carbon dots (CDs) for environmental remediation has gained awareness because of the diverse economically viable and environmental friendly green precursors generated from biowastes and biomass compared to the toxic inorganic quantum dots and CDs prepared from chemical precursors. This review presents the recent progress in green CDs, including their synthesis methods and sensing applications for the detection of heavy metal ions such as Iron (III), Mercury (II), Copper (II), Chromium (VI), Lead (II), Arsenic (III), Cobalt (II), Aluminum (III), Silver (I), and Gold (III) which are prominent environmental pollutants. The comparison based on selectivity, sensitivity, quantum yield, detection limit, linear concentration range, and sensing mechanisms are also reported. This review also covers the performance of doped green CDs using heteroatoms, toward the detection of heavy metal ions. Apart from the future perspectives, this review provides a general guide to use such environmental friendly CDs to detect harmful pollutants.

## Graphical abstract

**Figure:**
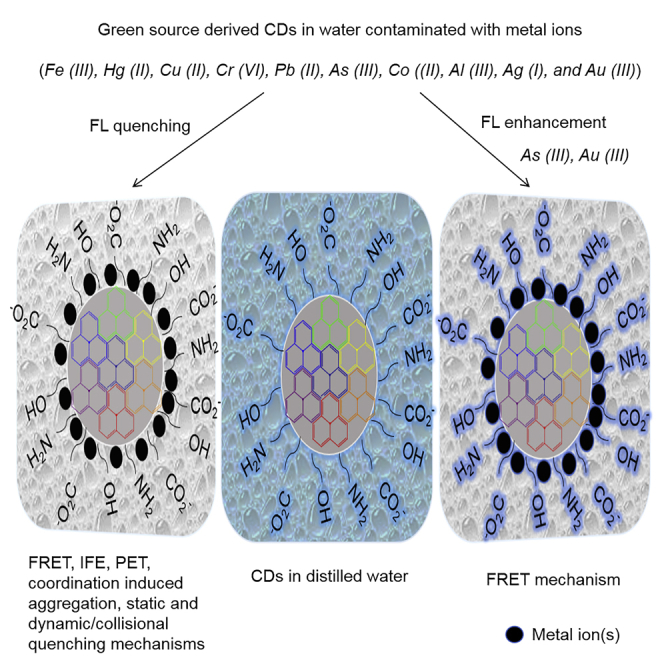

## Introduction

Incorporation of toxic contaminants and pollutants into the human body, either directly through drinking water, food, or absorption through the skin, has been a serious issue in recent years. Although toxicity of contaminants may depend on their characteristics, such as size, dosage, and exposure time (Bogireddy et al., 2021b), their escalating exposure to the human body has impacted the public and individual health adversely. Among the several nanomolecular level toxic contaminants, heavy metal ions, such as Iron (III), Mercury (II), Copper (II), Chromium (VI), Lead (II), Arsenic (III), Cobalt (II), Aluminum (III), Silver (I), Gold (III), and organic pollutants are all considered to be scourge for human health because of their extensive presence in the aqueous form in pharmaceutical, textile, and agricultural wastes (Bogireddy et al., 2019; Bogireddy et al., 2020b). Several internationally approved standard toxic limits are available from the World Health Organization (WHO) and the Environmental Protection Agency (EPA) for the safety evaluation of contaminants (Bogireddy et al., 2021b). Owing to the necessity to identify the contaminant(s) concentration at extreamly low level, using highly selective and sustainable probe techniques (Bogireddy et al., 2019), and their evaluation with respect to the standard permissible limits, can be proven beneficial for toxicity testing. In this scenario, several nanotechnology based detection systems have been proposed recently (Xu et al., 2020). Various nanomaterials, including carbon-based and metallic nanoparticles, have provided a potential solution for these existing environmental challenges (de Marco et al., 2019; Latha et al., 2020; Bogireddy et al., 2020a; Long et al., 2021). Among them, green synthesized carbon dots have attracted attention because of their sustainability, low cost, biocompatibility, and ease of use.

**Figure 1:**
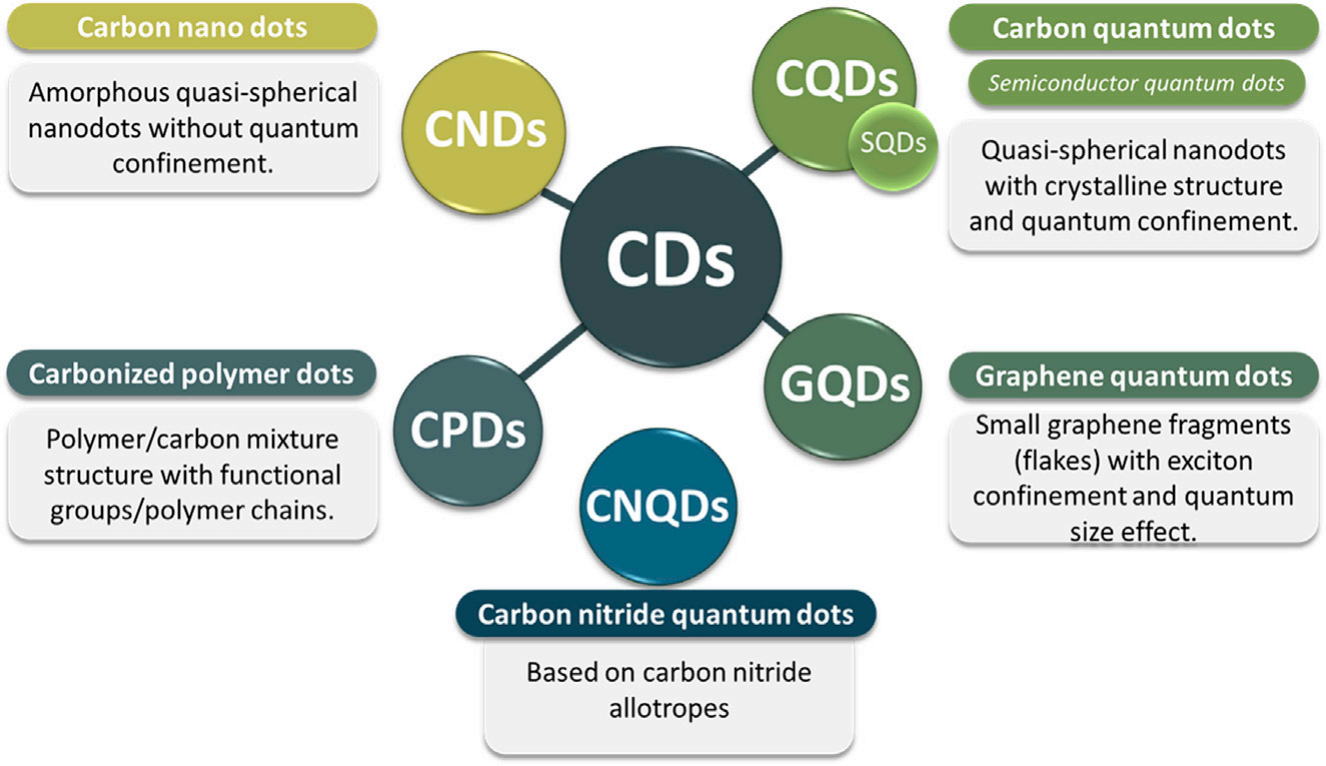
Carbon dots classification is based on the composition, structure, and dimensions (Cayuela et al., 2016; Li et al., 2021a; Semeniuk et al., 2019; Xia et al., 2019; Zhu et al., 2015).

Carbon dots (CDs or C-dots) is a generic name used for several nanosized carbon materials (Zhu et al., 2015). Xu et al. (2004) first unintentionally obtained fluorescent CDs during the purification process of single-walled carbon nanotubes (Xu et al., 2004), and later, their surface passivation resulted in an increased emission in 2006 (Sun et al., 2006), followed by the generation of Graphene Quantum Dots (GQDs) in 2008 (Ponomarenko et al., 2008). Besides having the intrinsic fluorescence property, generally, one of the dimensions of CDs is below 10 nm (Zhu et al., 2015), although some CDs as large as 60 nm (Semeniuk et al., 2019) have also been reported. Although classification and nomenclature for carbon nanomaterials is still a topic of debate, based on the characteristics and properties, different groups have classified CDs (Cayuela et al., 2016) into the following four major categories: Graphene Quantum Dots (GQDs), Carbon Nanodots (CNDs), Carbon Quantum Dots (CQDs), and Carbonized Polymer Dots (CPDs). GQDs are small graphene fragments with a single or few graphene sheets (size below 10 nm) to induce exciton confinement and quantum size effect. Although the size-dependent bandgap of GQDs is very similar to Semiconductor Quantum Dots (SQDs), the interaction between sheets confers to GQDs' superior properties, such as thermal and electrical conductivity (Li et al., 2019a; Xia et al., 2019; Yan et al., 2019; Zhao et al., 2020). CNDs are amorphous quasi-spherical dots without quantum confinement. In the amorphous graphitic $sp^{2}$ structures of CNDs, the near-UV to blue emission could be due to the recombination of the photogenerated electron-hole pairs (Shamsipur et al., 2018). CQDs are zero-dimensional quasi-spherical shaped crystalline nanoparticles with ultrasmall sizes (less than 10 nm) with quantum confinement effects (Cayuela et al., 2016; Murugan and Sundramoorthy, 2018; Li et al., 2019b; Semeniuk et al., 2019; Kováčová et al., 2020). The amorphous shell over sp^2^/sp^3^ conjugated core contains several functional groups with oxygen (from 5 to 50 wt %., depending on the synthesis method) (Lim et al., 2015; Zhang and Yu, 2016). Carboxylic acid moieties at the surface increase water solubility, whereas functionalization with other heteroatoms improves fluorescence property (Baker and Baker, 2010). CPDs consist of a polymer/carbon mixture structure with several functional groups/polymer chains, both inside and/or on the surface of the carbon core (Zhu et al., 2015;Xia et al., 2019). CPDs are classified into four subgroups, two with carbonized cores (as in CNDs or CQDs), third subgroup being one with a polycrystalline carbon structure, and the fourth being a highly dehydrated cross-linking and close-knit polymer frame (Xia et al., 2019). Most of the CDs synthesized by bottom-up methods have been considered CPDs, especially when there is incomplete carbonization of polymeric clusters (Song et al., 2017). The CPDs' stability is better than polymers because of carbonization (Tao et al., 2019). The CPDs can also be obtained by decorating the CNDs or CQDs with polymers or organic molecules (Xia et al., 2019). In addition, recent studies suggest a new category of Carbon Nitride Quantum Dots (CNQDs) (Li et al., 2021a) (Figure 1).

**Figure 2:**
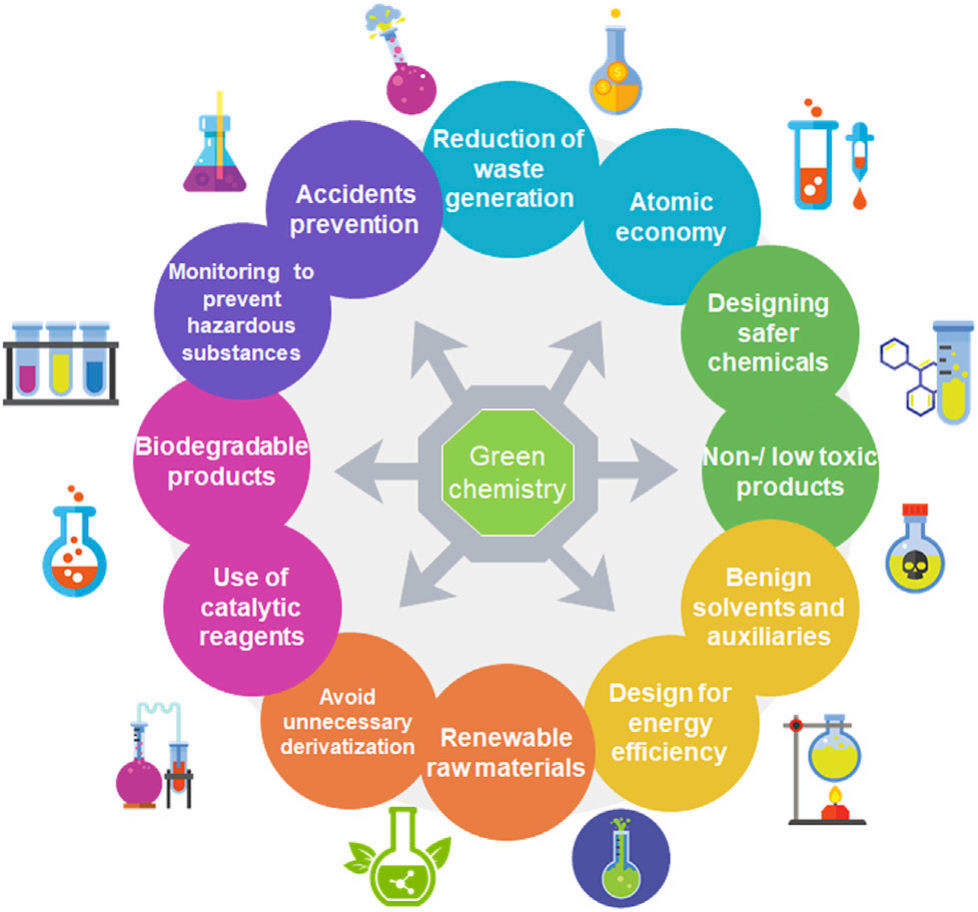
Twelve principles of Green Chemistry are based on the minimization of toxic solvents and non-generation of residues (Adapted from de Marco et al., 2019).

Compared with the traditional SQDs (CdSe, WO_3−x_, CdS, etc.), the CDs possess strong luminescence and small sizes and superior photostability against blinking and photobleaching, less toxicity, high conductivity, excellent aqueous solubility, biocompatibility, and chemical stability. They are produced using relatively easy, cost-effective, and eco-friendly preparation methods (Devi et al., 2019; Li et al., 2019a; Xia et al., 2019; Liu et al., 2020c). Hence, these unique characteristics enable a broad range of applications in bioimaging and biological labeling (Devi et al., 2019; Molaei, 2019), drug delivery (Li et al., 2020; Su et al., 2020) photocatalysis (Han et al., 2020; Seng et al., 2020), electrocatalysis (Liang et al., 2020; Xiao et al., 2020), sensing (Jiang et al., 2020; Wen et al., 2020), and photovoltaics (Gao et al., 2020; Wen et al., 2020). On the other hand, one can avoid using or producing hazardous materials during the synthesis process by adopting the twelve main principles of green chemistry. These principles are multifaceted and propose environmentally favorable techniques (de Marco et al., 2019), such as the use of dyes from plants (Roy Maulik, 2019) for the reduction of hazards solvents and non-generation of residues (Figure 2).

## Biomass

**Figure 3:**
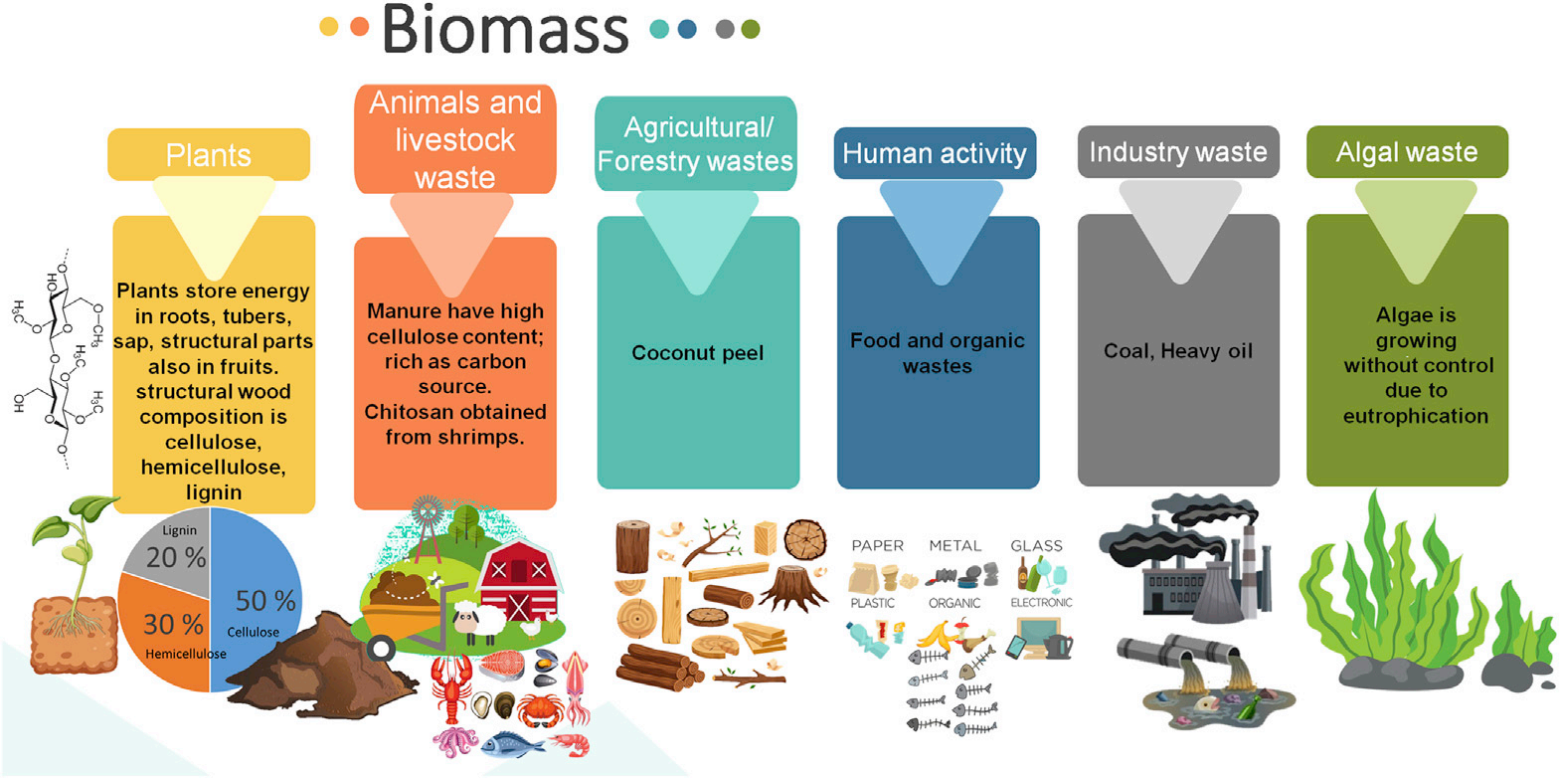
Main sources of biomass include plants and wastes from livestock, agriculture to algal (Borghei et al., 2018; da Rosa and Ordóñez, 2022; Barbosa et al., 2015; Gao et al., 2021; Kang et al., 2020; Qi et al., 2019; Zhang et al., 2017, p. 201).

“Biomass” is defined as biological material from anything alive or was alive a short time ago, whereas “waste” is any material required or intended to discard. It is an extensive, complex, surplus, heterogeneous, biodegradable, and bioorganic material that can be extracted from a vast number of sources (Kang et al., 2020; Gao et al., 2021), such as plants (da Rosa and Ordóñez, 2022), animals waste, agricultural, and forestry wastes, livestock wastes (Barbosa et al., 2015), by-products from operations in the industry (Gao et al., 2021), human activity waste (Kang et al., 2020), and algal waste coming from eutrophication (Zhang et al., 2017) (Figure 3).

Although biomass is mainly composed of carbon, oxygen and hydrogen, minor levels of some heteroatoms such as nitrogen, sulfur, phosphorus, alkali, and heavy metals are also present (Tang et al., 2021). The natural presence of heteroatoms in biomass derived precursors saves the additional steps related to the incorporation of external heteroatoms (Meng et al., 2019, p. 20). The abundance of functional groups in biomass structure provides multiple pathway reactions (Liu et al., 2020b; Das et al., 2021). If it comes from agricultural by-products, it generally contains an average composition of 40–50% cellulose, 20–30% hemicellulose, 20–25% lignin, and 1–5% ash (Kang et al., 2020; Mahat and Shamsudin, 2020). In adddition, it could be classified as having natural or anthropogenic origin (Pang et al., 2020).

Algae based biomass is a resource to obtain nitrogen in carbon based nanomaterials (Guo et al., 2017), where its presence can improve the QY of CDs (Abu-Ghosh et al., 2017). Ramanan et al. reported algal blooms based CDs (size 8nm) with 13% QY (Ramanan et al., 2016) and Singh et al. has demonstrated their use as nonylphenol nanosensor (Singh et al., 2020b). CDs from algal biomass have been obtained by hydrothermal treatment (Amjad et al., 2019) and applied as H_2_O_2_ sensors as well (Zhang et al., 2019a).

In addition to the conventional use of biomass in generating fuels and for feedstock (for industrial and animal uses) (da Rosa and Ordóñez, 2022), instead of chemically synthesized carbon compounds, biomass is presently being extensively researched as a possible carbon source to form CDs and cellulose colorimetric sensors (Qi et al., 2019)(Guo et al., 2019).

Although the synthesis of CDs from renewable bio-sources has its own challenges, it has been a desirable approach (De and Karak, 2013), because of several advantages, like sustainability, low cost, added value to waste, pollution-free materials, and green preparation methods (Liu et al., 2019c; Liu et al., 2019b; Singh et al., 2019; Yu et al., 2019; Ahmadi et al., 2020; Boruah et al., 2020; Wang et al., 2020a; Liu et al., 2020a; Lu et al., 2020; Das et al., 2021; Ding et al., 2021; Lou et al., 2021). Moreover, biomass-derived nanomaterials exhibit excellent water solubility, nontoxicity, and good biocompatibility (Wang et al., 2020b; Luo et al., 2021; Krishnaiah et al., 2022).

**Figure 4:**
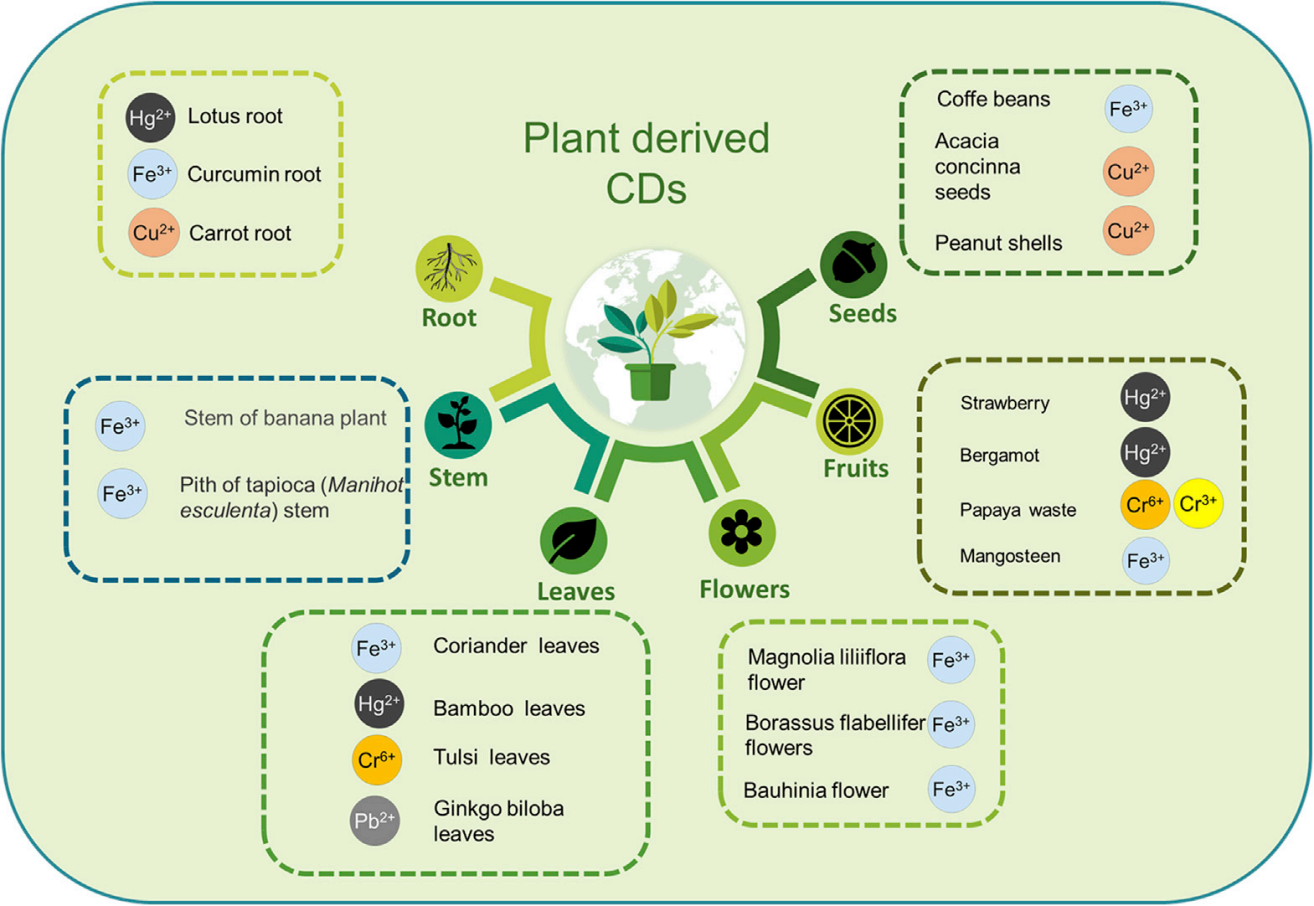
Some examples of CDs derived from different parts of the plant (Bhatt et al., 2018; Gu et al., 2016; Huang et al., 2013; Lim et al., 2015; Liu et al., 2019d; Sachdev and Gopinath, 2015; Singh et al., 2019; Xu et al., 2018; Yang et al., 2017; Yu et al., 2015; Zulfajri et al., 2019).

Among their several applications, numerous CDs derived from plant parts have been used to sense metal ions in water (Figure 4), such as Lotus root for Hg(II) (Gu et al., 2016), Strawberry for Hg(II) (Huang et al., 2013), Bamboo leaves for Pb(II) and Hg(II) (Liu et al., 2014), Bergamot for Hg(II) (Yu et al., 2015), Papaya for Cr(III), Cr(VI) (Pooja et al., 2019), Tulsi leaves for Cr(VI) (Bhatt et al., 2018), *Ginkgo biloba* leaves for Pb(II) (Xu et al., 2018), Coriander leaves for Fe(III) (Sachdev and Gopinath, 2015), and Mangosteen for Fe(III) (Yang et al., 2017).

The present study reviews the development of CDs synthesized from green precursors, toward the detection of heavy metal ions from 2010 onwards. Various synthesis methods, along with their advantages and limitations, relevant to the environment are also outlined. The recent progress in applications of green CDs to detect heavy metals such as Iron (III), Mercury (II), Copper (II), Chromium (VI), Lead (II), Arsenic (III), Cobalt (II), Aluminum (III), Silver (I), and Gold (III), along with their sensing mechanisms is reviewed in detail. We hope this review article can help in the future utilization of green CDs for optical sensing of various environmental pollutants.

## Synthesis techniques for obtaining CDs

**Figure 5:**
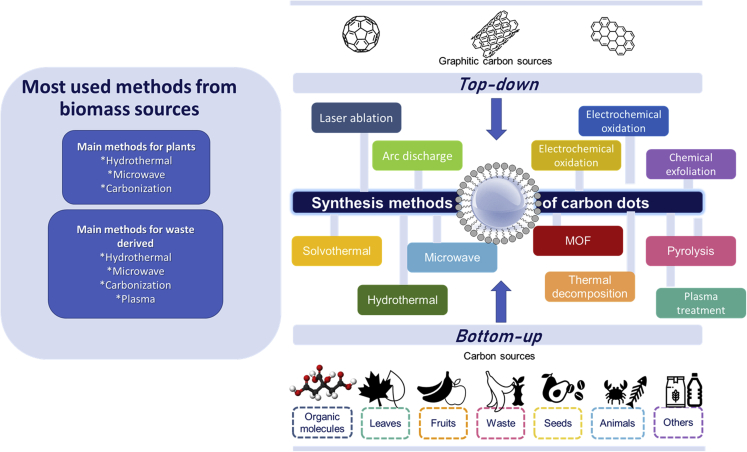
Major synthesis techniques and their classification

The synthesis techniques are principally divided into two classes: top-down and bottom-up methods (Figure 5). Generally, GQDs are obtained from top-down chemical synthesis procedures, whereas CQDs are obtained by bottom-up techniques (Lim et al., 2015).

### Top-down approach

The top-down approach involves the transformation of larger carbon structures into nano-size fragments through some form of energy. The synthesis methods under this approach include laser ablation (Xu et al., 2004; Yatom et al., 2017; H. Ramirez et al., 2019), arc discharge (Dey et al., 2014), and electrochemical oxidation (Bao et al., 2011), which involve the synthesis of CDs from carbon sources (large-sized graphene membranes, carbon nanotubes, graphite, commercially available activated carbon). These methods imply harsh experimental conditions, expensive equipment, and specific steps, thus limiting their practical and scalable applicability (Ma et al., 2012; Kang et al., 2020).

Although arch discharge is the most widely used method to synthesize CNTs (Herrera-Ramirez et al., 2019) and a variety of carbon nanostructures (Yatom et al., 2017), it also produces nanomaterials during the purification process of single-walled carbon nanotubes (Xu et al., 2004). Two cylindrical graphite electrodes, mounted vertically/horizontally inside the chamber filled with Helium or Argon gas, are ignited when brought close together. A gap of 1–2 mm is maintained for steady discharge. High-energy (4000–6000K temperature) plasma is produced because of the electric current, resulting in carbon vapors drifting toward cathode (He et al., 2016; Cao et al., 2019), leading to the formation of CQDs (Ludmerczki et al., 2019).

On the other hand, chemical oxidation involves no specialized equipment and is used for large-scale production. Qiao et al. (Qiao et al., 2010), reported that carbon materials with amorphous structures are used as precursors for the preparation of CDs because of their more accessible breakdown compared to bulk carbon allotropes with periodic structures. Moreover, easily carbonized and dehydrated materials can be used as raw materials to prepare CDs. The quantum yield of CDs prepared by this method is around 0.43–14.7 % (Qiao et al., 2010; Calabro et al., 2018). In addition, the electrochemical oxidation process is carried out using different electrode materials (graphite rods, carbon nanotubes, carbon paste, carbon fiber, and platinum sheets) in electrolytic solutions, and changing the applied potential has been shown to tune the size of CDs electrochemically (Bao et al., 2011).

Furthermore, laser ablation utilizes pulsed lasers (Nd: YAG) with high pulse energy, focused on a target in liquid/gas, resulting in the generation of plasma (Thongpool et al., 2012). During the laser ablation process, the local high pressure and high temperature can directly cut a target into microparticles/nanoparticles without residues, avoiding the introduction of impurities and contamination (Cui et al., 2020). The occurrence of photothermal vaporization or Coulomb explosion mechanism minimizes the solid content's size, controlled by laser fluence and pulse duration. The external chemical agents are not required in this method and do not generate by-products, thus guaranteeing a high-purity synthesis (Doñate-Buendia et al., 2018).

On the other hand, an easy and short-time ultrasonic technique not only has been used to break the large carbon materials by the action of very high energy of ultrasonic sound waves but also has also been found helpful in making CDs from small molecules. For example, Wang et al. (Wang et al., 2020a) synthesized N-doped CDs from ascorbic acid and ammonia, Ma et al. (Ma et al., 2012) using glucose and aq. ammonia, Dang et al. (Dang et al., 2016) obtained white fluorescence CDs (applied as white LEDs) using oligomer polyamide resin as carbon precursor and ethylenediamine as passivant. The as-prepared CDs were well dispersed, and had low crystallinity and functional groups at the surface (Seng et al., 2020).

### Bottom-up approach

As the bottom-up pathway is attractive due its simple application and easily tunable properties (Chahal et al., 2021), it has been the main approach to obtain CDs (da Júnior et al., 2021). Here the small size components (organic molecular precursors) are self-assembled by physical or chemical processes (Kashani et al., 2019; Baragau et al., 2021; Li et al., 2021b) such as hydrothermal, solvothermal, pyrolysis, combustion, microwave-assisted, or electrochemical process to promote the interaction and the formation of CDs (Shaker et al., 2020; Deng et al., 2021; Hou et al., 2021; Xu et al., 2021). Among them, the foremost techniques for biomass-derived CDs have been the hydrothermal/solvothermal method and pyrolysis. Hydrothermal/solvothermal carbonization is predominantly used to synthesize CDs because of its low-cost, eco-friendly, and nontoxic processes (Yang et al., 2017; Bogireddy et al., 2019; Latha et al., 2020; Bogireddy et al., 2020b; Bogireddy et al., 2020a; Anuar et al., 2021; Ge et al., 2021). In addition, water soluble carbon dots are generally made from hydrothermal and microwave techniques (Kang et al., 2020). The hydrothermal method uses water as a solvent in which the precursors are carbonized in an aqueous solution at high temperatures and vapor pressures to prepare water-soluble CDs. For instance, reactions have been done with green precursors like coriander (Sachdev and Gopinath, 2015), mangosteen (Yang et al., 2017), onion (Bandi et al., 2016), pseudo stem of banana (Vandarkuzhali et al., 2017), etc. The obtained CDs generally possess an amorphous structure and relatively high quantum yield (Wang and Hu, 2014; Choi et al., 2018).

The solvothermal synthesis involves the usage of various solvents such as ethanol and N, N-dimethylformamide (DMF) (Wang and Hu, 2014; Choi et al., 2018), using green precursors, e.g., bamboo leaves (Liu et al., 2019d), honey (Yang et al., 2014), and corn bract (Zhao et al., 2017). Another practical, economical, green, and fast method for synthesizing CDs is through microwave technique consisting of electromagnetic radiation-induced molecular transformations/polymerization (generally around 2.45 GHz). Its efficiency is based on the ability of polar molecules to absorb microwave radiation and transform it into heat by dielectric heating (Bogireddy et al., 2021b), where polarity decides the formation of hydrophilic, hydrophobic, or amphiphilic CDs based on an interaction between precursors and solvents (de Medeiros et al., 2019). Some CDs reported using microwave technique with green carbon precursors are lotus roots (Gu et al., 2016), potato dextrose agar (Gupta et al., 2016), table sugar (Ansi and Renuka, 2018), quince fruit powder (Ramezani et al., 2018), green natural kelp (Zhao et al., 2019a), and flour (Qin et al., 2013). Although some CDs could be synthesized via microwave (using organic solvents), they have found limited use in biomedical applications because of certain toxicity levels, solid state synthesis in microwave of CDs has been demonstrated to be relatively more effective and less toxic (He et al., 2016; Cao et al., 2019).

In addition, similar to microwave synthesis, thermal decomposition is also a cost-effective, fast, and easy to operate standard bottom-up technique to synthesize CDs. In this technique, a compound is chemically decomposed by the action of heat. The reactions involved in this method are primarily endothermic. There are two types of decomposition reactions, namely irreversible (involving proteins, starch) and reversible (involving limestone, ammonium chloride) (Sharma and Das, 2019). Typically, bottom-up methods imply usage of organic molecular or polymeric precursors (single or multicomponent) (Hutton et al., 2017). Most of the CDs from citric acid (CA) are obtained by thermal decomposition in which CA is dehydrated and reduced in the range of 180–200°C. It has been observed that this procedure involves the formation of different intermediates and not all of them are precursors for the synthesis of fluorescent CDs (Kashani et al., 2019; Wang et al., 2019). Similar low-cost, scalable, and eco-friendly pyrolysis technique consists of an irreversible thermal decomposition reaction where an inert atmosphere, very high temperature, and controlled pressure is used for the decomposition of organic materials resulting in solid residue containing a higher content of carbon (Stan et al., 2015; Esfandiari et al., 2019; Kashani et al., 2019; Sharma and Das, 2019) and is an efficient way to fabricate high-performance CDs from human urine (Essner et al., 2016), pigeon feathers (Roshni and Ottoor, 2015), coconut milk (Roshni and Ottoor, 2015), sago waste (Tan et al., 2014), peanut shells (Ma et al., 2017), and papaya waste (Pooja et al., 2019).

Furthermore, plasma treatment is a one-step physical method that introduces functional groups onto the raw materials as well as the synthesized CDs (Park et al., 2017). Surface oxidation or passivation of CDs is necessary to generate fluorescence and make them hydrophilic (Wang et al., 2012). The concentration of precursor and operating voltages during plasma treatment affect the composition and size distribution of CDs resulting in the difference of PL emission (Ma et al., 2019). A typical plasma treatment system consists of a radio-frequency generator, an electrode, dielectric materials, a ceramic substrate, a diffuser, a sample stage, a gas inlet/outlet, and a vacuum system. Argon and oxygen/nitrogen are used as the carrier gas and reactive gas, respectively (Park et al., 2017).

### Other methods

Many more novel methods have emerged to synthesize CDs on a large scale. These methods include sand bath, one-step refluxing, and magnetic hyperthermia. The sand bath method is also an economically viable, environmentally friendly, facile, and green approach for the large-scale synthesis of CDs. Gudimella et al. used the sand bath strategy to obtain CDs from citrus fruit peels. These CDs exhibited excellent photoluminescence properties and multicolor fluorescence (Gudimella et al., 2021). One-step refluxing process has also been developed to obtain multicolor CDs (blue, green, and yellow) with the precursor solution, L-cysteine/D-(+)-galactose, and different concentrations of NaOH refluxed at 80°C for 24 h (Wang et al., 2017). Using the similar method, CDs have also been prepared using edible carrots as a carbon source and an aqueous solution of trisodium phosphate as a catalyst (Jayanthi et al., 2019).

Magnetic hyperthermia (MHT) is a new green strategy for synthesizing CDs for large-scale production. This technique is employed as a heating source capable of increasing energy generation and spreading under an electromagnetic field. The magnetic nanoparticles can generate heat and thus activate many chemical reactions. The particle properties and the value of the Curie temperature determine the efficiency. CDs based on the MHT technique, can be used as the coating ink to construct color-converting fluorescent films. Zn^2+^, Na^+^, and K^+^-doped CDs were synthesized using this technique (in only 1 h) with a quantum yield of about 50% and were used as nanofillers for improving the mechanical performance of the electronic spinning derived polycaprolactone nanofibrous scaffold (Zhu et al., 2020).

The main advantages and limitations of the various synthesis techniques are outlined in Table 1.

**Table 1:** Main advantages and limitations of synthesis routes adopted for the production of carbon nanoparticles

Method	Advantages	Disadvantages	Synthesis route
Laser ablation	• Tunable Surface states • Easily Controllable morphology and size • high purity • Good reproducibility (Wang and Hu, 2014; Atchudan et al., 2017; Li et al., 2019c)	• Low quantum yield • High cost • Complicated operation • limits large-scale production (Li et al., 2019c)	Top-down
Arc discharge	• Feasible to generate doped graphene carbon dots (Dey et al., 2014)	• undesirable carbon material generated • requiring purification • Large particle size distribution under the condition of high acidity, high potential, and high energy (Mishra et al., 2018; Wang et al., 2019; Pan et al., 2020)	Top-down
Electrochemical oxidation	• Controllable size • High purity • Good reproducibility • Process under the normal temperature and pressure (Ming et al., 2012; Z. Li et al., 2019c; Pan et al., 2020)	• difficult to control • complex purification process (Pan et al., 2020).	Top-down
Hydrothermal	• High purity • Good dispersion • Ease in particle size control • Nontoxic (Lou et al., 2021)	• High vapor pressure (1 MPa ∼ 1 GPa) (Zhang and Yu, 2016).	Bottom-up
Solvothermal	• Products are formed slowly • CDs properties influenced by the kind of solvent (Lou et al., 2021)	• Low yield and purity, unsatisfactory uniformity of product size, and morphology (Lou et al., 2021).	Bottom-up
Microwave	• Homogeneous temperature distribution • Direct heat of the target molecules • lower reaction temperatures • Possibility of very fast solid-state synthesis (Cao et al., 2019; de Medeiros et al., 2019; Lan et al., 2020).	• Use of small reactors limit the large-scale production (de Medeiros et al., 2019)	Bottom-up
Thermal decomposition	• Easy to operate • Less time consuming • Low cost • Viability for large-scale production (Sharma and Das, 2019)	• Non fluorescent intermediates could be formed (Ludmerczki et al., 2019)	Bottom-up
Pyrolysis	• Simple process • Economical • Feasible for mass production of highly emissive CDs (Lai et al., 2012)	• High temperature is required • Difficult to separate CDs and other small molecules of the raw materials (Lai et al., 2012).	Bottom-up
Plasma treatment	• Easy and low-cost process • Able to generate functional groups on the surface of CDs by reactive gas plasma • No additives are required • Works at room temperature • One-step and large-scale viability (Park et al., 2017)	• Requires reactors with special configuration (Ma et al., 2019)	Bottom-up

## Characterization

All the carbon nanomaterials have been characterized using some conventional/basic techniques accompanied by few advanced characterizations representing the main landmarks for determining the nature of the material.

### Optical characterization

Among the conventionally used basic characterization, absorption characteristics are analyzed for the identification of the electronic transition bands with the help of UV-visible absorbance spectroscopy (Papaioannou et al., 2018). The PL emission and excitation spectra are measured using a fluorescence spectrometer and are the basic parameters needed for the optical sensing in carbon-based nanomaterials (Papaioannou et al., 2018; Rigodanza et al., 2021). The absolute/relative quantum yield is measured using integrating sphere/comparing the fluorescence intensity with another sample (generally a dye) with known quantum yield (Würth et al., 2013). To identify the material luminescence properties like photoluminescence, chemiluminescence, and phosphorescence, the lifetime measurements are performed using PL lifetime spectroscopy (Sun et al., 2020). The first approximation for the particle size measurements have been achieved using dynamic light scattering (Javed and O’Carroll, 2021) based particle size analyzers, where the Brownian motion of the nanoparticles scatters the laser light and the analysis of the fluctuations is interpreted. Surface charge of the carbon dots has been obtained from zeta potential studies.

### Structural and morphological characterization

The crystallinity and graphitic nature of the carbon dots can be identified using X-ray diffractometer (XRD) (Bogireddy et al., 2021a). X-ray photoelectron (XPS) (Reagen et al., 2021) and Fourier transform infrared spectroscopy (FT-IR) experiments have been performed to analyze carbon dots' chemical and structural composition (Bogireddy et al., 2020a). The chemical structure, phase, and molecular interactions of carbon dots have been analyzed from Raman Spectroscopy studies. For the advanced characterization of carbon nanoparticle systems, such as averaged particle sizes, shapes, distribution, and surface-to-volume ratio, small/wide-angle X-ray scattering (SAXS and WAXS) measurements have been performed (Papaioannou et al., 2018; Rigodanza et al., 2021). The structure of a material can be identified by using Nuclear Magnetic Resonance (NMR) measurements through the interaction of nuclear spins under a powerful magnetic field (Arroyave et al., 2021).

The size, shape, and crystallinity of carbon dots have been obtained from high resolution-transmission electron microscope and selected area (electron) diffraction (SAED) images (Bogireddy et al., 2020a). Moreover, the topography of carbon dot materials has been obtained from atomic force microscopy (AFM) characterization (Guo et al., 2015; Bhati et al., 2018).

## Carbon dots for heavy metal ion detection

Some heavy metal ions, such as copper, iron, aluminum, and chromium (III), are nutritionally essential and required by some organisms, but higher concentrations can cause toxicity (Xu et al., 2018; Bogireddy et al., 2019; Bogireddy et al., 2020b; Bogireddy et al., 2021b). Although in trace amounts, heavy metal ions such as chromium (VI), lead(II), arsenic(III), cadmium(II), and mercury(II) are the most common nonbiodegradable and toxic pollutants in industrial effluents (Bhatt et al., 2018). Environmentally sound and practically feasible sensors for detecting heavy metal ions are critical for minimizing water pollution and preventing harmful effects at the outset. The development of efficient and real-time sensors for detecting contaminants in living systems and the entire environment is still being researched (Sachdev and Gopinath, 2015). Metal-based nanoparticles and organic dyes are being phased out in favor of fluorescent nanocarbons to detect harmful contaminants.

Many green CDs have been used for sensitive and selective colorimetric and fluorometric metal ion sensing because of surface oxygen moieties which in turn are responsible for coordinating with metal ions which results in the PL quenching. The energy transfer between nano carbons and metal ions through selective interactions because of functional groups and surface traps are the main parameters responsible for the PL quenching (Sachdev and Gopinath, 2015; Bogireddy et al., 2019; Bogireddy et al., 2020b; Bogireddy et al., 2021b). All the characteristics, such as surface functionalities, edge structure, size, and morphology of nanocarbons, have an impact on selectivity. Furthermore, CDs' optoelectronic properties, stability, and applications can be controlled by doping them with nitrogen, boron, sulfur, and phosphorus (Miao et al., 2020).

For designing a fluorescence (FL) sensor, different detection mechanisms such as Förster/fluorescence resonance energy transfer (FRET), photoinduced electron transfer (PET), inner filter effect (IFE), coordination induced aggregation, FL quenching, etc. have been used, and are based on either attenuation or enhancement of fluorescence. Stokes first discovered that IFE is an important mechanism to improve the detection sensitivity of fluorescent sensors by converting absorption signals into fluorescence readout. IFE demands a spectral overlap between the donor molecules’ emission and the acceptor molecules’ absorption. Moreover, it does not require crucial covalent interaction between sustainable CDs and pollutants. FRET effect is identified from the decrease in the change in the FL lifetime measurement of the receptor’s in the presence of CDs (Choudhury et al., 2017). PET is a redox reaction in which an excited electron is transferred from CDs to the receptor. In the static and dynamic quenching process, the former involves FL quenching between the acceptor and donor, whereas dynamic/collisional quenching refers to the transfer of an electron from the donor to the acceptor (Chatzimarkou et al., 2018).

This article covers a variety of natural precursors for the synthesis of carbon-based nanoprobes that have been used as photoluminescent nanoprobes to detect toxic metal ions such as Copper (II), Iron (III), Aluminum (III), Chromium (III), Chromium (VI), Lead (II), Arsenic (III), Cadmium (II), Silver (I), Mercury(II), Cobalt(II) and Gold(III). The main parameters that have been used for the evaluation of the CDs are limit of detection (LOD), quantum yield (QY), and linear concentration of range (LCR), which indicates the over all efficiency and the operational range of green CDs toward the corresponding metal ion sensitivity (Bogireddy et al., 2019; Bogireddy et al., 2021b).

### Fe (III)/Fe (II)

Iron is essential for life and exists in ferrous (II) and ferric (III) ionic states (Shander et al., 2009). Out of two oxidation states, Fe (III) is more harmful because of insolubility and production of toxic radicals. An excess amount of Fe (III) in the environment and the human body is the primary factor for the diseases such as Parkinson, cytotoxicity, metabolic disorders, etc. The tendency to accept electrons from surroundings by free or excess iron ions may damage cellular systems (Singh et al., 2020a). As per World Health Organization (WHO) guidelines, the maximum permissible limit of Fe (III) in drinking water is 5.36 μM (World Health Organization, 2004). As Fe (III) detection using a selective, cost-effective, and sensitive device is required, the development of simple and portable fluorescent CDs for detecting Fe (III), has generated a lot of interest (World Health Organization, 2004; Wang et al., 2016; Atchudan et al., 2017). The main works describing Fe (III) sensing through green precursor generated CDs have been tabulated (Table 2) below.

**Table 2:** Detection of Iron (II) / Iron (III) using green precursor derived CDs

Precursor (Year)	Synthesis Technique	Limit of Detection (LOD), μM	Linear concentration range (LCR), μM	Quantum yield (QY), %	Metal ions screened for selectivity
*Coriander leaves* (Sachdev and Gopinath, 2015) (plant Source)	Hydrothermal	0.4	0–60	6.48	Co (II), Hg (II), Fe (II), **Fe (III)**, Ag (I), Ca (II), Pb (II), Mg (II), Cd (II), Zn (II), Pb (II), Ni (II), and Cu (II)
*Honey* (Yang et al., 2014) (animal source)	Solvothermal	1.7 × 10^−3^	5.0 × 10^−3^1×10^2^	19.8	Hg (II), Fe (II), **Fe (III)**, Pb (II), Ag (I), Ca (II), Co (II), Mn (II), Sr (II), Zn (II), K (I), Na (I), and Cu (II)
*Mangosteen pulp* (Yang et al., 2017) (plant source)	Hydrothermal	52 × 10^−3^	0–0.18 ×10^9^	–	Na (I), K (I), Mg (II), Ca (II), Cr (III), Co (II), Ni (II), Cu (II), Zn (II), Ag (I), Hg (II), Cd (II), Pb (II), Fe (II), Al (III),and **Fe(III)**
*Onion waste* (Bandi et al., 2016) (plant source)	Autoclave	0.31	0–20	28	Na (I), K (I), Mn (II), Ba (II),Fe (II), Cu (II), Sn (II), Cr (III), Al (III), Pb (II), Ni (II), Mg (II), Zn (II), Hg (II), Cd (II), Ca (II), and **Fe(III)**
*Pseudostem of banana* (Vandarkuzhali et al., 2017) (plant source)	Hydrothermal	6.4 × 10^−3^	0–100	48	Ag(I), Mn(II), Co(II), Fe(II), Cu(II), Cr(III), Al(III), Pb(II), Ni(II), Mg(II), Zn(II), Hg(II), Cd(II), Ca(II), and **Fe (III)**
*Sugarcane molasses* (Huang et al., 2017) (plant source)	Hydrothermal	1.46	0–20	5.8	K(I), Ca(II), Mg(II), Cd(II), La(III), Pb(II), Mn(II), Co(II), Cr(III), Fe(II), Cu(II), and **Fe (III)**
*Sweet potato* (Shen et al., 2017 (plant source)	Hydrothermal	0.32	1–100	8.64	**Fe (III)**, Ag(I), Hg(II), Cr(III), Co(II), Al(III), Cd(II), Cu(II), Ni(II), Ba(II), Ca(II), Pb(II), Mn(II), and Zn(II)
*Mangifera indica leaves* (Singh et al., 2020a) (plant source)	Pyrolysis	3.12^a^	–	18.2	Cu (II), Al (III), Mg (II), K (I), Li (I), Na (I), Mn (II), Zn (II), Co (II), Ni (II), Cd (II), Sn (II), and **Fe (II)**
*Tomato* (Kailasa et al., 2019) (plant source)	Chemical Oxidation	0.016 (B- CDs)	0.1–2.0	12.70	Ba (II), Ca (II), Cu (II), Hg (II), Zn (II), Ni (II), Fe (II), Al (III), and **Fe (III)**
		0.072 (G- CDs)		4.21	
		0.065 (Y- CDs)		2.76	
*Coffee beans* (Zhang et al., 2019b) (plant source)	Hydrothermal	15.4 × 10^−3^ 16.3 × 10^−3^ (up-conversion)	0–0.10 ×10^9^	–	K (I), Na (I), Mg (II), Zn (II), Ag (I), Cd (II), Ca (II), Pb (II), Co (II), Ni (II), Hg (II), Al (III), Cu (II), Fe (II), and **Fe (III)**
*Soybeans* (Zhao et al., 2019b) (plant source)	Ultrasonic	2.9	0–30	16.7	Ca (II), Cd (II), Co (II), Cu (II), Cr (III), **Fe (III)**, Fe (II), Hg (II), Mn (II), Na (I), Pb (II), and Zn (II)
*Syringa oblata Lindl* (Diao et al., 2018) (plant source)	Hydrothermal	0.11	0.5–80	12.4	Ag (I), Na (I), K (I), Cd (II), Cr (III), Co (II), Cu (II), Ca (II), Fe (II), Hg (II), Mg (II), Mn (II), Zn (II), Ni (II), Pb (II), **Fe (III),** and Al (III)
*Lycii Fructus* (Sun et al., 2017) (plant source)	Hydrothermal	21 × 10^−3^	0–30	17.2	K (I), Li (I), Na (I), Zn (II), Ca (II), Cd (II), Cu (II), Mn (II), Pb (II), Mg (II), Co (II), Hg (II), Fe (II), Cr (III), Al (III), and **Fe (III)**
*Rice residue + glycine* (Qi et al., 2019) (plant source)	Hydrothermal	0.7462	3.3–32.2	23.48	Ni (I), Ag (I), Cd (II), La (III), Pb (II), Co(II), Ce (III), K (I), Na (I), Ca (II), Y (III), Zr (IV), Al (III), Mg (II), Cu (II), Hg (II), **Fe (III)**, and Fe (II)
D*warf banana peel + aq. ammonia* (Atchudan et al., 2020) (plant source)	Hydrothermal	0.66	5–25	23.0	Al (III), Ca (II), Cd (II), Co (II), Cr (III), Cu (II), **Fe (III)**, Hg (II), Mn (II), Ni (II), Pb (II), and Zn (II)
*Piper betle (Betel) leaf* (Atchudan et al., 2019) (plant source)	Hydrothermal	0.43	5–30	–	Al (III), Ca (II), Cd (II), Co (II), Cr (III), Cu (II), **Fe (III)**, Hg (II), Mn (II), Ni (II), Pb (II), and Zn (II)
*Phyllanthus acidus (P*. *acidus) +aq. ammonia* (Atchudan et al., 2018) (plant source)	Hydrothermal	0.9	2–25	14	Al (III), Ca (II), Cd (II), Co (II), Cr (III), Cu (II), **Fe (III)**, Hg (II), Ni (II), Pb (II), and Zn (II)
*Bombyx mori silk+Citric acid* (Liu et al., 2017) (animal source)	Hydrothermal	0.38	0.5–4	61.1	Ag (I), Al (III), Ca (II), Cd (II), Co (II), Cu (II), Cr (III), Hg (II), K (I), Mg (II), Pb (II), and **Fe (III)**
L*emon juice + aq. ammonia* (Mondal et al., 2016) (plant source)	Hydrothermal	2.5	1–90	–	Na (I), Al (III), Mn (II), Ag (I), Ni (II), Co (II), Zn (II), Cd (II), Hg (II), Mg (II), Pb (II), Cu (II), and **Fe (III)**
*Chionanthus retusus (C*. *retusus) fruit extract* (Atchudan et al., 2017) (plant source)	Hydrothermal	70	0–2	9	Al (III), Ca (II), Cd (II), Co (II), Cr (III), Cu (II), Fe (II), **Fe (III)**, Hg (II), Ni (II), Pb (II), and Zn (II)
*Kiwi fruit peels* + *ammonium hydroxide* (Atchudan et al., 2021) (plant source)	Hydrothermal	0.95 and 0.85	5–25	14 19	Al (III), Ca (II), Cd (II), Co (III), Cr (III), Cu (II), **Fe (III)**, Hg (II), Mn (II), Ni (II), Pb (II), and Zn (III)
*Betel leaves* + *aq.ammonia* (Atchudan et al., 2019) (plant source)	Hydrothermal	0.135	0.3–3.3	4.21	K (I), Cu (I), Na (I), Pb (II), Cu (II), Cr (VI), Zn (II), Fe (II), Cd (II), Ag (I), **Fe (III)**, Hg (III), and Mn (III)
*Curauá (Ananas erectifolius) fibers* (Raja et al., 2021) (plant source)	Hydrothermal	0–30	0.77	–	**Fe (III)**, Na(I), K (I),Mg (II), Ca (II), Ba (II), Cr (II), Mn (II), Fe (II), Co (II), Ni (II), Cu (II), Ag (I), Zn (II), Cd (II), Hg (II), and Al (III)
*Ripe banana peels+*ethylene diamine + *L-cysteine* (Das et al., 2021) (plant source)	Hydrothermal	10–200 ×10^−6^	121 × 10^−6^	27	**Fe (III)**, Hg (II), Cu (II), Cr (VI), Cd (II), Mg (II), Na (I), and K (I)
*Biomass waste (orange peel*, *ginkgo biloba leaves*, *paulownia leaves, and magnolia flower)* (Wang et al., 2020a) (mixture of 4 different plant sources)	Hydrothermal	0.088 (MF-CQDs)	0.2–100	8.13	Ag (I), K (I), Na (I), Pb (II), **Fe (III)**, Fe (II), Zn (II), Cu (II), Mg (II), Ca (II), Al (III), Cd (II), Cr (III), Ba (II), and Hg (II)
		0.073 (OP-CQDs)		4.29	
		0.080 (GB-CQD)		7.72	
		0.10 (PL-CQDs)		4.74	

Commonly used salt for Fe(III) detection was FeCl_3_ and for Fe(II) was FeCl_2_ (Selectivity marked in bold letters; last column).

a Denoted values were recalculated for uniformity in the corresponding units with respect to other reports.

Briefly, Abhay Sachdev et al. (Sachdev and Gopinath, 2015), hydrothermally synthesized the fluorescent CDs from coriander leaves without using additional passivating agents for surface modification and showed that the proposed CDs have pH-dependent optical response toward Fe (III) sensing. Under acidic (pH < 7) and higher basic conditions (pH > 9), because of protonation of surface carboxylic groups, low quenching efficiency was observed. This resulted in the weaker interactions in the CD–Fe (III) complex, and instead of CDs, the complexation of Fe (III) through hydroxyl groups occurred. Within pH range seven to nine, because of the deprotonation of surface carboxylic groups, the observed higher quenching efficiency was attributed to the strengthening of Fe (III) - CDs interaction. Xiaoming et al. (Yang et al., 2014) also proposed the possible understanding of fluorometric sensing of Fe (III) using solvothermal technique generated honey mediated CDs in 2014. In this study, several reaction conditions were optimized, such as pH (6.0), time (10 min), temperature (35 °C), and Britton–Robinson buffer for sensing Fe (III) effectively. An increase in the particle size of CDs, observed in the presence of Fe (III) (HR-TEM analysis), confirmed the CD-Fe (III) coordination induced aggregations which in turn caused the FL quenching of CDs.

Several other groups used Mangosteen pulp (Yang et al., 2017), sugarcane molasses from industrial waste (Huang et al., 2017), Sweet potato (Shen et al., 2017), and Syringa oblata Lindl (Diao et al., 2018) as precursors to obtain fluorescent CDs for detection of Fe (III). They (Huang et al., 2017; Shen et al., 2017; Yang et al., 2017; Diao et al., 2018) attributed the PL quenching to the electron transfer between Fe (III) and carboxyl/hydroxyl groups present around the CDs, which led to the coordination-induced aggregation of CDs in the presence of Fe (III). The CDs obtained from mangosteen pulp were able to detect the sunset yellow in LCR of 0–60 μM because of FL quenching (Yang et al., 2017), whereas CDs obtained from sweet potato were able to detect Fe (III) in living cells (Shen et al., 2017). Mild interference in fluorescence intensity, upon the additional incorporation of Fe(II), Co(II), Cr(III), and Cu(II), was also reported (Shen et al., 2017). In highly luminescent CDs using onion waste, through FL lifetime measurements, concluded that dynamic FL quenching involved the transfer of electrons from the excited state of CDs to the vacant orbital of Fe (III), resulting in non-radiative electron-hole recombination (Bandi et al., 2016). CDs synthesized using pseudo-stem of the banana plant exhibited fluorescent “turn-off” sensing and high selectivity toward Fe (III) in the presence of other ions (Vandarkuzhali et al., 2017). The CDs synthesized from *Mangifera indica* leaves showed excellent binding capability with Fe (II) ions and were able to detect Fe (II) ions in water and a Linogen tablet (as a real sample). The electron transfer process between Fe (II) and CDs has been reported (Singh et al., 2020a). Using H_2_SO_4_ and H_3_PO_4_ as oxidizing agents, blue-fluorescent, green-fluorescent, and yellow-fluorescent CDs were synthesized from tomato (Solanum Lycopersicum). Apart from the better Fe (III) detection performance observed through blue-CDs, all the CDs were used as FL probes for sensing Fe (III) in biofluids and pharmaceutical samples. The LODs of the method were lower than the permissible limits of Fe (III), recommended by the US Environmental Protection Agency (EPA) ( Bogireddy et al., 2021b). In addition, electron transfer induced FL quenching through time-resolved FL, Zeta potential, HR-TEM, and FTIR studies in the proposed CDs + Fe (III), are also reported (Kailasa et al., 2019).

The hydrothermally prepared CDs using coffee beans (Zhang et al., 2019b) revealed the down-conversion and up-conversion fluorescence measurements. The characteristics of CDs were studied in the presence of Fe (III) using TEM, EDS, absorbance, and FL lifetime measurements to identify the possible mechanism of aggregation-induced electron transfer. The Lycii Fructus obtained CDs (Sun et al., 2017) were able to detect Fe (III) in the Yellow River water sample, urine samples, and living HeLa (Henrietta Lacks) cells. Through enhanced absorbance and FTIR/XPS data, jointly lead towards the inner filter effect and photoelectron/energy transfer being responsible for the change in the optical response of the CDs in the presence of Fe (III). The low toxicity and high detection limit of bright blue fluorescent nano-biomass dots (NBDs) synthesized from soybean (Zhao et al., 2019b) indicated their possible applications in biological and environmental systems. Contrary to Xiaohan Sun et al. (Sun et al., 2017), although Wen-Bo Zhao et al. (Zhao et al., 2019b) reported no absorbance change in the presence of Fe (III), for better understanding, the FL lifetime measurements suggested electron transfer as the possible mechanism behind the quenching of optical signal of CDs in the presence of Fe (III). No interference studies were reported with other metal ions for the detection of Fe (III) (Sachdev and Gopinath, 2015; Shen et al., 2017; Sun et al., 2017; Zhang et al., 2019b).

Nitrogen-doped carbon quantum dots (N-CDs) prepared using rice residue and glycine, revealed specificity toward Fe (III) detection in real water samples. The quenching of fluorescence of N-CDs in the presence of Fe (III) was because of the special coordination between phenolic hydroxyl groups on the surface of N-CDs and Fe (III). The N-CDs were also applied in detecting TCs, i.e., tetracycline, terramycin, and chlortetracycline with LODs as 0.2367, 0.3739, and 0.2791 μM, respectively (Qi et al., 2019). Similarly, in the case of NCDs produced from the dwarf banana peel (biowaste) with aqueous ammonia, the interaction between surface functionalities (−COOH, −OH, and –NH_2_) of CDs with Fe (III) was found to be responsible for the FL quenching. In addition, because of high QY, N-CDs were employed in bioimaging applications, health care, environmental protection, and as fluorescent ink (Atchudan et al., 2020).

CDs synthesized using Piper betel (Betel) leaf (both as a carbon and nitrogen precursor) (Atchudan et al., 2019) reported that Fe (III) and CDs interaction may be because of the electron transfer process. In fluorescent nitrogen-doped CDs prepared by *Phyllanthus acidus* (as carbon source) with aqueous ammonia (nitrogen source) (Atchudan et al., 2018), FL quenching was attributed to the strong affinity between the CDs and Fe (III) (Atchudan et al., 2018). Both types of CDs can be used as an alternative for the traditional fluorescent inks because of their excellent fluorescence stability, pollution-free, easily washable, and biocompatibility (Atchudan et al., 2018, 2019). The CDs synthesized using *Bombyx mori* silk natural fibers and citric acid reported that FL quenching might be because of the interactions between Fe (III) and the hydroxyl groups on the surface of CDs, which resulted in the aggregation of CDs (Liu et al., 2017). Fluorescent NCQDs synthesized using lemon juice and ammonia showed FL quenching behavior by Fe (III) using both static and dynamic quenching mechanisms. NCQDs were used to detect Fe (III) in environmental water samples and biological applications (Mondal et al., 2016). In N-CDs synthesized using *Chionanthus retusus* (*C. retusus*) fruit extract (carbon precursor) and aqueous ammonia (nitrogen source), FL quenching could be related to the non-radiative electron transfer process between the N-CDs and Fe (III). These N-CDs can also be used in the early detection of yeast infections in biological samples (Atchudan et al., 2017).

Two FL CDs were prepared using leftover kiwi fruit peel without and with ammonium hydroxide (NH_4_OH). Reduced fluorescence intensities of the CDs (to nearly zero) were attributed to the complex formation because of the stronger affinity of CDs with Fe (III) ions (Atchudan et al., 2021). N-CDs made from betel leaves and ammonia revealed blue fluorescence and exhibited good selectivity and sensitivity to picric acid and Fe (III). The fluorescence is quenched because of a strong interaction between Fe (III) and the surface groups of N-CDs (Kalanidhi and Nagaraaj, 2021).

Using a hydrothermal technique, CDs made from common and inexpensive biomass waste (Orange peel, *Ginkgo biloba* leaves, Paulownia leaves, and Magnolia flower) displayed homogeneous particle size, excellent water solubility, high stability, and equivalent optical characteristics. High sensitivity and selectivity were achieved by using biomass CQDs as fluorescence sensors to detect Fe (III) ions. The fluorescence quenching induced by CDs aggregation (in the presence of Fe (III)) was categorized as static quenching (Wang et al., 2020a). The LOD of Fe (III) in the above cases was much lower than the permitted value given by WHO, showing that the green CDs can detect metal ion Fe (III). All the above CDs, based upon fluorescent sensing systems, show many advantages, including rapid detection, high sensitivity, and good selectivity toward detection of Fe (III) with a wide linear response range.

### Hg (II)

Depending on its oxidation states, Mercury exists as Hg(0) Hg(I) and Hg(II) in our surroundings (water and air) (Kim and Zoh, 2012). Hg (II) is a nonbiodegradable, highly toxic metal ion for living organisms and the environment (Huang et al., 2013; Zhao et al., 2017; Zulfajri et al., 2019). A slightest accumulation in food chains, especially in aquatic systems, leads to serious health hazards to human beings because of its poisoning effect on the kidney, liver, cardiovascular, and central nervous system (CNS) of the human body (Gu et al., 2016). The guidelines by WHO for intake of mercury through water is 1 μg/L (Liu et al., 2019a) and via air is 2 μg/kg body weight per day. Therefore, it is necessary to detect Hg for protecting the environment and avoiding health hazards. Many FL probes have been reported for the detection of Hg (II) ions using metal nanoparticles, semiconductor quantum dots, carbon nanoparticles, and some biomaterials (Gu et al., 2016). Green CDs are preferred over the above mentioned inorganicfluorescent probes because of their complex synthesis mechanisms, toxic agents, and high cost. Table 3 describes the green CDs' main characteristics that have been used so far for Hg (II) detection.

**Table 3:** Detection of Mercury (II) using green precursor derived CDs

Precursor (year)	Quantum yield (%)	Synthesis Technique	Limit of Detection (LOD), nM	Linear Concentration Range, μM	Metal ions screened for selectivity
*Corn bract leaves* (Zhao et al., 2017) (plant source)	6.90	Solvothermal	9	0–40	Na (I), K (I), Mg (II), Ca (II), Ba (II), Al (II), Sn (II), Pb (II), Cr (II), Mn (II), Fe (III), Fe (II), Co (II), Ni (II), Cu (II), Zn (II), Ag (I), Cd (II), and **Hg (II)**
*Bamboo leaves* (Z. Liu et al., 2019d) (plant source)	–	Solvothermal	0.22	0.001–1	Al (III), Fe (III), Cr (III), Cu (II), Mg (II), Pb (II), Zn (II), Ca (II), Cd (II), Mn (II), Co (II), Ag (I), Na (I), K (I), **Pb (II)**, and **Hg (II)**
*Lotus root* (Gu et al., 2016) (plant source)	19.0	Microwave	18.7	0.1–60	**Hg (II),** Cd (II), Cu (II), Pb (II), Mg (II), Fe (III), Sr (II), Ca (II), Ba (II), Al (III), Fe (II), Co (II), and Zn (II)
*Strawberry Juice* (Huang et al., 2013) (plant source)	6.3	Hydrothermal	3	0.001–50	Ca (II), Ag (I), Ni (II), Cr (III), Al (III), Cu (II), Ba (II), Fe (III), Pb (II), Zn (II), Fe (II), Co (II), Mg (II), Mn (II), Cd (II), and **Hg (II)**
*Pomelo peel* (Lu et al., 2012) (plant source)	6.9	Hydrothermal	0.23	–	Ag (I), Ca (II), Cd (II), Co (II), Cu (II), Fe (II), Mg (II), Mn (II), Ni (II), Pb (II), **Hg (II),** and Zn (II)
*China grass carp scales* (Liu et al., 2019a) (animal source)	19.9	Microwave supported Hydrothermal	14	0.014 -30	Ag (I), Ba (II), Bi (II), Cd (II), Co (II), Cr (III), Cu (II), Fe (III), Fe (II), **Hg (II)**, Mo (VI), Mn (II), Ni (II), and Pb (II)
*Flour* (Qin et al., 2013) (plant source)	5.4	Microwave	0.5	0.0005–0.01	Zn (II), Pb (II), Ni (II), Ca (II), Mg (II), Cu (II), Co (II), Cd (II), Fe (II), **Hg (II),** and Mn (II)
*Human Hair* (Guo et al., 2016) (human source)	10.7	Thermal	10	0–1 ×10^3^	Ag (I), Al (III), Ba (II), Ca (II), Cd (II), Co (II), Cr (III), Cu (II), Fe (III), **Hg (II)**, Mg (II), Mn (II), Ni (II), Pb (II), and Zn (II)
*Honey* (Srinivasan et al., 2016) (animal source)	–	Hydrothermal	1.02	0–10 ×10^−3^	**Hg (II),** Pb (II), Cu (II), Cd (II), Ni (II), Co (II), Cr (III), Ag (I), Al (III), Fe (III), Au (III), Mg (II), Mn (II) and Zn (II)
*Highland barley+ethanediamine* (Xie et al., 2019) (plant source)	14.4	Hydrothermal	480	10–160	Cu (II), Mg (II), Co (II), Zn (II), Mn (II), Cd (II), Ca (II), Pb (II), Ba (II), and **Hg (II)**
*Pigeon* (Ye et al., 2017) (animal source)	24.8 (*Feathers*)	Pyrolysis	10.3	0–1.2	Ag (I), Ca (II), Cd (II), Co (II), Al (III), Cu (II), **Hg (II)**, K (I), Pb (II), Mg (II), Mn (II), Na (I), Ni (II), **Fe (III)**, Pd (II), Ba (II), Ce (III), and Zn (II)
	17.4 (*Egg white*)		34.6	0.05–1.2	
	16.3 (*Egg yolk*)		34.9	0–1.6	
*Coconut Milk* (Roshni and Ottoor, 2015) (plant source)	–	Thermal Pyrolysis	16.5	30.5 × 10^−3^	Pb (II), Ni (II), Co (II), Cd (II), **Hg (II)**, Zn (II), Mn (II), and Fe (II)
*Pineapple Peel* (Vandarkuzhali et al., 2018) (plant source)	42.0	Hydrothermal	–	0.1–100	Ag (I), Al (III), Co (II), Cd (II), Cr (II), Cu (II), Ca (II), Fe (II), Fe (III), Pb (II), Mn (II), Mg (II), Ni (II), Zn (II), and **Hg (II)**
*Cucumber juice* (Wang et al., 2014) (plant source)	–	Hydrothermal	180	1–70	**Hg (II)**, Cu(II), Ca(II), Mg(II), Cd(II), Pb(II), Mn(II), Fe(III), Ba(II), Al(III), Ni(II) and Co(II)
*Jinhua Bergamot* (Yu et al., 2015) (plant source)	50.7	Hydrothermal	5.5	0.01–100	Ag (I), Al (III), Ca (II), Co (II), Cr (III), Cu (II), Mg (II), Mn (II), Ni (II), Pb (II), Zn (II), Fe (II), **Hg (II)** and **Fe (III)**
*Hongcaitai* (Li et al., 2018) (plant source)	12.1	Hydrothermal	60	0.2–15	**Hg (II)**, Na (I), K (I), Ca (II), Mg (II), Cr (III), Co (II), Cd (II), Mn (II), Cu (II) , Zn (II), Fe (III), Fe (II) , Al (III), Pb (II), Ni (II), and Ag (I),
*Muskmelon* (Desai et al., 2019) (plant source)	26.9	Acid oxidation	330	1.0–25	**Hg (II),** Cu (II), Co (II), Ni (II), As (III), and Cr (III),
*Chinese Yam* (Li et al., 2015a; 2015b) (plant source)	9.3	Hydrothermal	1.26	10–30 ×10^−3^	**Hg (II),** Ag (I), Au (III), Pb (II), Cu (II), Al (III), Fe (III), Ce (III), Ba (II), Ca (II), Cd (II), Co (II), Mg (II), Mn (II), Ni (II), Sn (IV), Zn (II), Cr (III), and K (I)
*Human Urine* (Essner et al., 2016) (human source)	14	Pyrolysis	2.7	0–45	Zn (II), Sr (II), Ba (II), Mn (II), Ca (II), Sn (II), Ni (II), Cu (II), Pd (II), Hg (II), **Fe (III), Cu (II), Pd (II),** and **Hg (II)**
*Lemon Juice* (Gharat et al., 2019)	2.4	Hydrothermal	36 ×10^3^	0–1.82 ×10^3^	**Hg (II)**, Ag (I), Au (III), Pb (II), Cu (II), Al (III), Fe (III), Ce (III), Ba (II), Ca (II), Cd (II), Co (II), Mg (II), Mn (II), Ni (II), Sn (IV), Zn (II), Cr (III), and K(I)
*Dunaliella salina* (Singh et al., 2019) (algal source)	8	Hydrothermal	18	0.03–0.20	Na (I), K (I), Fe (II), Fe (III), Cr (III), Ni (II), Cu (II), Zn (II), Cd (II), Hg (II), Mn (II), Mg (II), Co (II), As (II), Pb (II), Ag (I) ,**Hg (II)** and **Cr (VI)**

Commonly used salts for Hg(II) detection were Hg(NO_3_)_2_ and HgCl_2_ (Selectivity marked in bold letters; last column).

The first report on Hg (II) sensing was presented by Wenbo Lu et al. (Lu et al., 2012) using CDs derived from Pomelo peel waste in environmental water samples. In this work, the possible FL quenching mechanism was stated as the electron transfer process, which was confirmed by removing Hg (II) from the CDs surface with chelator addition to CDs-Hg (II) aqueous solution. Later, Hong Huang et al. (Huang et al., 2013) synthesized luminescent nitrogen-doped (6.88%) carbon nanoparticles from strawberry juice, tested in environmental water samples, reported that the obtained FL quenching was a dynamic/ultrafast electron transfer process from the considerable change in the fluorescence lifetime values. Slight interference by Cu(II) was also observed.

Chunfeng Wang et al. (Wang et al., 2014) prepared water-soluble N/S/P co-doped FL CDs from Cucumber juice (size less than 10 nm) (Wang et al., 2014). Roshini V. et al. (Roshni and Ottoor, 2015) prepared CNPs using Coconut milk, Jing Yu et al. (Yu et al., 2015) prepared water-soluble fluorescent CDs with Jinhua bergamot and Ye et al. (Ye et al., 2017) prepared CDs using Pigeon feathers, Egg white, and Egg yellow. They all explained that the FL quenching might be because of easing non-radiative electron/hole recombination through the charge transfer process. This process occurs among Hg (II) and carboxyl/hydroxyl groups present on the surface of CDs (Roshni and Ottoor, 2015; Yu et al., 2015; Ye et al., 2017).

Zhuo Li et al. (Li et al., 2015b) synthesized nitrogen-doped CDs (size 2.7 ± 1.4 nm) using Chinese yams for the detection of Hg (II) in real water samples. It is reported that fluorescence intensity increases because of the addition of Hg (II) analyte and the formation of a hairpin structure. Jingjin Zhao et al. (Zhao et al., 2017) reported a nanohybrid dual emission sensor from CDs prepared using Corn bract and anhydrous ethanol using a solvothermal process having emission bands around 470 and 678 nm. These results indicated the ratiometric Hg (II) sensing through an electron-rich aromatic ring containing porphyrin. Hg (II) recoveries in human serum samples and river water samples indicated that proposed ratiometric sensors might be introduced in health and environment related applications. Jeremy B. Essner et al. (Essner et al., 2016) did thermal upcycling of collected urine and synthesized luminescent CDs which could be used as nanoprobes in aqueous solutions for the detection of Hg (II). They described the complete recovery of FL signal by adding EDTA to Hg (II) quenched samples. Srinivasan et al. (Srinivasan et al., 2016) developed honey-based MoS_2_ nanosheets/DNA/carbon dots nano-assembly to detect Hg (II) in the environmental samples. Lu Shuang Li et al. (Li et al., 2018) prepared CDs using Hongcaitai (*Brassica campestris* L.var.purpurea Bailey), a vegetable fromChina, and was tested in river water. FL quenching was ascribed to its contact with sulfur-containing groups on the surface of CDs through the electron transfer process. Liu et al. ( Liu et al., 2019d) prepared multi-emission FL nanohybrids CDs using extracts from bamboo leaves to detect Hg (II) in complex environmental water such as river water. LOD was lower than the safe limits of the heavy metal ion in drinking water as recommended by the WHO. FL quenching confirms a formation of Hg (II) porphyrin complex, indicating a static quenching process. The microwave-assisted nitrogen (5.23%) doped multicolor fluorescence bioimaging (Gu et al., 2016) through CDs prepared from Lotus root has been reported in addition to their application in testing environmental water samples. They tried to understand possible Hg (II) sensing processes using optical measurements. No change in the lifetime measurement but drastic change (absence) in the absorption peak of CDs was observed in the presence of Hg (II), which was attributed to the CDs-Hg (II) complex formation.

Guanhong Liu et al. (Liu et al., 2019a) prepared CDs from China Grass carp scales (CGCS) raw material and gave more insight into FL quenching mechanisms using FTIR, lifetime, and absorbance studies. FL quenching of CDs was identified as fluorescence resonance energy transfer and weresuccessfully tested in water samples and cosmetics.

Water-soluble photoluminescent CDs of flour (purchased from the local market) were successfully tested against real lake water samples and found to be very stable, without any precipitated or floating nanodots for several months, reported that Hg (II) might have a stronger affinity toward the carboxylic group on the surface of CDs than other metal ions. In addition, the PL intensity was reported to be pH-dependent (Qin et al., 2013). Yongming Guo et al. (Guo et al., 2016) thermally synthesized CDs from human hair (99% keratin and 1% other elements) into highly fluorescent CQDs sensors and were tested in tap water. A disappearance of absorbance peaks and a longer lifetime were observed with Hg (II) addition into CDs, which confirms both static and dynamic effects on the system. The slight interference by Ag(I), Cu (II), and Fe (III) as compared to other metal ions was also reported.

In Nitrogendoped CDs from Highland Barley (73.2% carbohydrates and 8.1% proteins ; abundant and inexpensive biomass), the FL quenching is attributed to the strong chelating ability of Hg (II) toward the carboxylic group on N-CDs surface (Xie et al., 2019). Pineapple peel based CDs could satisfactorily detect Hg (II) in tap water and lake water. The dynamic/electron transfer process indicated a decrease in the lifetime with Hg (II) addition to CDs (Vandarkuzhali et al., 2018). On the contrary, Desai et al. (Desai et al., 2019) prepared water-soluble multicolor emissive CDs using Muskmelon. They observed no change in the lifetime with Hg (II) addition to CDs, confirming a static quenching mechanism. In addition, they also tested these CDs for Hg (II) in biological and real water samples. CDs prepared by thermal decomposition of lemon juice were studied in an aqueous medium involving neutral reactants. The quenching of CDs by Hg (II) occured only at high pH conditions (Gharat et al., 2019). TheCQDs prepared using algal biomass-Dunaliella salina taken from Sambhar Lake from Rajasthan showed dynamic quenching. The possible FL quenching mechanism might be due to an electron transfer/dynamic process through the change in the lifetime of CDs in the presence of Hg (II) (Singh et al., 2019). No interference from other metal ions was reported (Lu et al., 2012; Gu et al., 2016; Li et al., 2018; Vandarkuzhali et al., 2018; Liu et al., 2019a; Singh et al., 2019).

Apart from the simple and scalable synthesis, low cost, excellent sensitivity/selectivity, as the limit of detection of Hg (II) in the majority of the above cases was much lower than the permitted value recommended by WHO (5 nM) and USEPA for Hg (II) analysis in real samples, all the CDs (mentioned above) prepared using green precursors may hold potential applications in environmental protection and water safety monitoring systems.

### Cu (II)

Copper produces a wide range of compounds, with oxidation states +1 and +2, commonly referred to as cuprous and cupric, respectively (Wiberg et al., 2001). Cu (II) is an essential element for living beings because the respiratory enzyme contains Cu (II) as a significant component of the complex cytochrome *c* oxidase. Exposure to high Cu (II) levels, even for a short time, can cause a gastrointestinal disturbance. In contrast, exposure for a longer duration is a reason for heavy damage to the liver and kidneys. The U.S. Environmental Protection Agency (EPA) has set up 1.3 ppm (20 μM) as a permissible safe limit in drinking water (Das et al., 2017). Optical sensors based on metal-based quantum dots (QDs) such as CdS, CdSe, ZnS, and CdSeTe, suffer from various limitations like time-consuming processes involving usage of environment threatening toxic heavy metals. Therefore, the development of new nontoxic optical sensors using green precursors derived CDs is the need of the hour. The main works describing Copper (II) sensing through green precursor generated CDs have been tabulated (Table 4) below.

**Table 4:** Detection of Copper (II) using green precursor derived CDs

Precursor (Year)	Quantum Yield %	Synthesis technique	LOD (μM)	Linear concentration range (μM)	Metals ion screened for selectivity
*Sago waste* (Tan et al., 2014) (plant source)	–	Pyrolysis	7.78	–	**Cu (II),** Cr (III), Co (II), Ni (II), Al (III), Ca (II), Pb (II), Zn (II), Sn (II), and Hg (II)
*Peanut shells* (Ma et al., 2017) (plant source)	10.58	Pyrolysis	4.8	0–5	**Cu(II)**, Ba (II), Na (I), Mn (II), K (I), Mg (II), Fe (III), Cd (II), Li (I), Al (III), Co (II), and Ca (II)
*Lemon juice+* *L-arginine* (Das et al., 2017) (plant source)	7.7	Thermal coupling	0.047	0- 15	**Cu(II),** Cd (II), Ba (II), Hg (II), Fe (II), Ag (I), Ca (II), Li (I), Mg (II), Pb (II), and Zn (II)
*Kelp +* *polyethylenimine* (Zhu et al., 2017) (algal source)	12.3	Hydrothermal	7 × 10^−3^ (CDs) 9 × 10^−3^ (CDs-FITC composites)	1–12.5	**Cu(II)**, Hg (II), Mn (II), Pb (II), Mg (II), Ca (II), Zn (II), Ba (II), Cd (II), Ni (II), Co (II), Fe (II), Fe (III), Na (I), K (I), Li (I), Ag (I), and NH_4_ (I)
*Grass* (Liu et al., 2012) (plant source)	4.2	Hydrothermal	1 × 10^−3^	0 -50	**Cu(II)**, Ag (I), Ba (II), Ca (II), Cd (II), Co (II), Fe (III), Hg (II), Mg (II), Mn (II), Ni (II), Pb (II) andZn (II)
*Bamboo leaves+ poly ethylenimine* (Liu et al., 2014) (plant source)	7.1	Hydrothermal	0.115	0–66	Co (II), Ca (II), **Cu (II),** Ni (II), Fe (III), Mn (II), Hg (II), Pb (II), Ba (II), Cd (II), **Hg (II), Co (II), Ni (II),** and **Fe (III)**
*Pu-erh tea* (Zhang et al., 2020) (plant source)	–	Brewing method	51 × 10^−3^	0–22	Al (III), Ba (II), Ca (II), Cd (II), Fe (II), Fe (III), Hg (II), K (I), Li (I), Mg (II), Mn (II), Na (I), Ni (II), Sr (II), Zn (II), and **Cu (II)**

Commonly used salts for Cu(II) detection were CuCl_2_, CuSO_4_ and Cu(NO_3_)_2_ (Selectivity marked in bold letters; last column).

The CDs prepared from Sago industrial waste have been used as an optical probe to sense Cu (II) in aqueous media. Compared to other metal ions, Cu (II) was reported with the highest absorption affinity toward the surface of CDs which might be the reason for FL quenching (Tan et al., 2014). Apart from having a relatively high QY, the CDs synthesized using peanut shells also used a scalable method to recycle peanut shells (Ma et al., 2017). Xiaohong Ma et al. (Ma et al., 2017) and Yuefang Hu et al. (Hu et al., 2017) attributed the FL quenching (with the addition of Cu (II)), to the presence of N and O functional groups on CDs surface. No interference from other metal ions was reported (Ma et al., 2017). Poushali Das et al. (Das et al., 2017) prepared NCDs using lemon juice with nitrogen content from l-arginine to detect Cu (II) in river water. The sensing mechanism was explored by adding EDTA to the quenched Cu (II)-CDs complex. The FL signal was restored entirely, which can eliminate the cupric amine complex's inner filter effect. X. Zhu et al. (Zhu et al., 2017) synthesized CDs using natural kelp and modified them with polyethyleneimine (PEI), and subsequently combined the CDs with fluorescein isothiocyanate (FITC) to generate CDs-FITC composites. They reported highly selective CDs for Cu (II) by amine groups from PEI followed by FL quenching via IFE. The slight intereference reported from Hg (II) was controlled by adding I^−^ in the aqueous suspension of CDs-FITC composites.

PPNDs (Photoluminescent Polymer Nanodots) using Grass have also been used to detect Cu (II) ions in real water samples. FL quenching of PPNDs was attributed to the energy transfer because of the chelation of Cu (II) ions with N and O of PPNDs. The fabricated N, O containing CDs have a faster chelating process and a higher thermodynamic affinity for Cu (II) than other metals (Liu et al., 2012). In branched polyethyleneimine (BPEI) capped bamboo leaves CQDs (BPEI-CQDs), PL quenching with a blue shift in BPEI CDs spectra (in the presence of Cu (II)) was observed. The quenching happens because of the detachment of BPEI from the surface of CDs. . In addition, Liu et al. used these CDs for Cu (II) detection in river water, showing their environmental water quality analysis (Liu et al., 2014).

Zhang et al. (Zhang et al., 2020) made CDs directly by combining Pu-erh tea with hot water (T-CDs). Using the acquired CDs and *o*-phenylenediamine (OPD), a new sensing universal platform is created that can be simultaneously applied as fluorescent and colorimetric dual-readout for Cu (II).

The limit of Cu (II) detection in all of the above cases was much lower than the permitted value recommended by WHO in drinking water. The prepared FL CDs and the precursors are eco-friendly, sustainable, scalable, and easy to prepare with high selectivity, good sensitivity, and fast Cu (II) detection in real samples.

### Cr (III) and Cr (VI)

The heavy metal, Cr (VI) is known for its toxic and carcinogenic nature and is used in different fields, like industrial, domestic, agricultural, medical, and technological (Li et al., 2011; Zhitkovich, 2011). The oxidation states of Cr (VI) and Cr (III) generally exist in the environment, where Cr (VI) (because of high solubility and mobility) causes long term adverse effects (Zou et al., 2006; Gu et al., 2012; Huang et al., 2021) and Cr (III) because of less solubility and mobility, is less toxic (Shinde et al., 2004; Jaishankar et al., 2014). As per WHO and BIS (World Health Organization, 2004), the permissible limit of Cr (VI) in drinking water is 0.05 mg/L (∼900 nM). Atomic absorption spectrometry Voltammetry, UV visible absorption spectrometry, inductively coupled plasma mass spectrometry, and X-ray fluorescence spectrometry have all been investigated for the speciation analysis of Cr in environmental samples (Pooja et al., 2019). In comparison to FL detection methods, although these methods are sensitive, they are unsuitable for industrial applications because of their complicated sample preparation processes, high equipment costs, and lack of timely and rapid detection. The green carbon quantum dots (CQDs) have received a lot of interest for their use in detecting Cr ions. The main works describing Cr(III)/Cr (VI) sensing through green precursor generated CDs have been tabulated (Table 5) below.

**Table 5:** Detection of Chromium III/(VI) using green precursor derived CDs

Precursor (Year)	Quantum yield (%)	Techniques Used	Limit of Detection, μM	Linear Concentration range, μM	Metal ions screened for selectivity
*Papaya waste (carica papaya)* (Pooja et al., 2019) (plant source)	23.7	Pyrolysis	2 × 10^−3^^a^	0.028–2.86^a^	Na (I), Li (I), Fe (II), Cu (II), Mg (II), Zn (II), Pb (II), Cd (II), Co (II), Ni (II), **Cr (III)**, **Cr (VI)**, Cr (III) + Cr (VI); **Cr (VI)** dominant over **Cr (III)**
*Pineapple Juice* (Sharma et al., 2017) (plant source)	10.06	Hydrothermal	0.052	0–18	Ag (I), Mg (II), Al (III), Cd (II), Cu (II), Fe (II), K (I), Na (I), Sr (II), Pb (II), Zn (II), and **Cr (VI)**
*Tulsi leaves* (Bhatt et al., 2018) (plant source)	3.06	Hydrothermal	0.015^a^	1.6–50	**Cr (VI)**
*Lemon Peel Waste* (Tyagi et al., 2016) (plant source)	14	Hydrothermal	73 × 10^−3^	2.5–50	Ni (II), Cd (II), Fe (II), Cu (II), Mn (II), Co (II), Ba (II), and **Cr (VI)**
*Black particulates of Petrol Soot* (Tripathi et al., 2016) (Hydrocarbon source)	–	Oxidation	0.51	0–20 ×10^3^	Na (I), Ba (II), Mn (II), Cd (II), Fe (II), Fe (III), As (III), Ni (II), Pb (II), Co (II), Cu (II), Zn (II), and **Cr (VI)**
*Denatured Milk* (Athika et al., 2019) (animal source)	–	Hydrothermal	14	–	Na (I), Eu (III), Sn (II), Ba (II), Pb (II), Cd (II), Ni (II), Cr (III), **Cr (VI)**, Al (III), Mg (II), Ag (I), K (I), Fe (II), Fe (III), Cu (II), Hg (II), Mn (II), and Co (II)
*Kelp* (Feng et al., 2019) (algal source)	20.5	Hydrothermal	0.52	0.01- 50	Mg (II), Ba (II), Cd (II), Hg (II), Fe (III), Cu (II), Mn (II), Zn (II), Al (III), Ag (I), Ca (II), Pb (II), and **Cr(VI)**
*Groundnuts + Ethylenediamine* (V et al., 2019) (plant source)	17.6	Hydrothermal	1.9	2–8	Ca (II), Cd (II), Co (II), Cr (III), **Cr (VI),** Fe (III), Cu (II), Mg (II), Ni (II), Pb (II), Hg (II), and Fe (II)
*Dunaliella salina* (Singh et al., 2019) ( algal source)	8	Hydrothermal	0.018	0.03–0.18	Na (I), K (I), Fe (II), Fe (III), Cr (III), Ni (II), Cu (II), Zn (II), Cd (II), Hg (II), Mn (II), Mg (II), Co (II), As (III), Pb (II), and Ag (I), **Hg (II) and Cr (VI)**
*Dried rose petals+* *ethylenediamine +L-cysteine* (Das et al., 2021) (plant source)	28	hydrothermal	81 × 10^−6^	10–200 ×10^−6^	Fe (III), Hg (II), Cu (II), Cr (III), **Cr (VI),** Cd (II), Mg (II), Na (I), and K (I)

Commonly used salts for Cr (VI) detection : K_2_Cr_2_O_7_, K_2_CrO_4_ and for Cr (III) detection : CrCl_3_ (Selectivity marked in bold letters; last column).

a Denoted values were recalculated for uniformity in the corresponding units with respect to other reports.

Pooja D. et al. (Pooja et al., 2019) developed EDTA functionalized CDs (size<10nm) of Papaya waste (*Carica papaya*) for Cr (III) and Cr (VI) sensing. The possible FL quenching process might be because of N and O containing functional groups on the surface of CDs and could detect both Cr (III) and Cr (VI) without any prereduction/oxidation step (Pooja et al., 2019). The spongy carbon nanoglobules (CNG) using Pineapple juice were synthesized for sensing highly hazardous metal ions. These CNGs exhibited hydrophilicity and stability for several weeks and sensing is attributed to the presence of –COOH, -OH, and –NH_2_ groups on the surface of CDs (Sharma et al., 2017). The Tulsi leaves-derived CDs (Bhatt et al., 2018) showed low toxicity, high fluorescence, and photostability. They were successfully applied for detecting Cr (VI) in spiked tap and industrial water, in addition to the recovery(of optical response of nanoprobes) ranging from 93–99% with the addition of ascorbic acid. FL probe using natural kelp CDs was prepared for testing in environmental water samples to determine chromium with sufficient recoveries (Feng et al., 2019). No interference studies in the presence of cations, anions, or multielement mixture were reported. Shreya Bhatt et al. (Bhatt et al., 2018), Shouai Feng et al. (Feng et al., 2019), and Mattath Athika et al. (Athika et al., 2019) (CDs prepared using denatured milk) explored mechanistic understanding using different optical characterizations. A good absorbance and FL emission spectral overlap, change in the zeta potential, and no change in CDs' lifetime was observed, which confirms the presence of a combination of IFE and static quenching. With an average dimension between 1 and 3 nm, the water-soluble lemon peel-derived CQDs possessed high photostability and tested for Cr (VI) in drinking water (Tyagi et al., 2016). The water-soluble photoluminescent graphene nanosheets using waste “black carbon particulates of pollutant petrol soot” were oxidized for making a clean environment (Tripathi et al., 2016). The possible FL quenching of CDs in the presence of Cr (VI) was attributed to the non-radiative recombination of electron-hole pairs because of low redox potentials and low-lying d-d transition states, including functional groups on the CDs' surface (Tripathi et al., 2016; Tyagi et al., 2016). N doped CDs prepared from ground nuts have not only exhibited enhanced quantum yield (17.6%) and high selectivity toward Cr (VI) as compared to undoped C-dots (7.8) (Roshni et al., 2019) but have also shown the potential to reduce Cr (VI) in the presence of glutathione (GSH) and humic acid.

The halophilic microalgae (Dunaliella Salina) derived nitrogen-phosphorus dual doped carbon dots have been applied in the detection of (on-off) Hg (II) and Cr (VI) in the live cell of a complex biological environment (; Bogireddy et al., 2021b). Interestingly, Singh et al. (Singh et al., 2019) observed absorbance and FL emission spectral overlap and change in the lifetime value, which signifies the involvement of both IFE and dynamic quenching in Cr (VI) sensing. In our experience, while detecting metal ions using CDs, Cr (VI) reduction to Cr (III), and subsequent PL quenching analyzed through XPS revealed the possible electron transfer from dual emissive CDs to Cr (VI) (; Bogireddy et al., 2021b), the detection limit of Cr (VI) in most of the above cases was much lower than the permitted value recommended by WHO in drinking water. Such CQDs provide remarkable advantages, including fast response, simplicity, and low instrumentation cost, and they are also promising candidates for the environmentally friendly and sustainable detection of Cr (VI).

### Pb (II)

Lead exists in three oxidation states: Pb(0), the metal, Pb(II), and Pb(IV). Pb(IV) is only formed under extremely oxidizing conditions and inorganic Pb(IV) compounds are not found under ordinary environmental conditions. Although organolead(II) compounds are known, organolead chemistry is dominated by the tetravalent (+4) oxidation state. Metallic lead (Pb (0)) exists in nature, but its occurrence is rare.

Lead (Pb) is a toxic d-metal (Tchounwou et al., 2012) known for causing harmful disorders in the human body. Although it exists in three oxidation states , it primarily occurs as Pb(II) in the environment (Abadin et al., 2007)**.** More than 5 μmol/L of Pb (II) concentration in blood can lead to diseases like mental disability, memory loss, anemia, migraine, and may even lead to death. The limit of Pb intake in drinking water as set by the Environmental Protection Agency (EPA) is less than 15.0 ppb (72 nM) (Chen et al., 2009; Huang et al., 2010; Li et al., 2010; Hou et al., 2011; Tchounwou et al., 2012; Feleafel and Mirdad, 2013; Gupta et al., 2016). Lead ions are found in drinking water because of Pb containing service pipes or/and other related accessories corroded in the presence of acidic water/water with low mineral content (Payne, 2008). Several DNAzyme sensors, aptamers, polymer dots, and inorganic nanomaterials such as gold, CdS, and ZnS have been devised in recent years to track and assess Pb (II) ions using fluorescence. But all the above methods suffer from limitations like usage of heavy metals and high cost. As a result, it is critical to develop simple and inexpensive materials that may be used as highly selective and sensitive probes to detect Pb (II) ions. Due to its green chemistry approach, the use of green precursors as carbon sources to synthesize CDs has sparked a lot of attention (Kumar et al., 2017). The main works describing Pb (II) sensing through green precursor generated CDs have been tabulated (Table 6) below.

**Table 6:** Detection of Lead (II) using green precursors derived CDs

Precursor (Year)	Quantum yield (%)	Techniques used	Limit of detection, nM	Linear concentration range, μM	Metal ions screened for selectivity
*Ocimum sanctum* *leaves (Tulsi Leaves)* (Kumar et al., 2017) (plant source)	9.3	Hydrothermal	0.59	10–1000	Cu (II), Mg (II), K (I), Ca (II), Ni (II), **Pb (II)**, Co (II), Hg (II), Cd (II), Na (I), Sn (II), and Al (III)
*Ginkgo biloba leaves* (Xu et al., 2018) (plant source)	16.1	Hydrothermal	0.055	0.1–20 × 10^−3^	Na (I), K (I), Co (II), Hg (II), Ag (I), Cu (II), Fe (II), Fe (III), Zn (II), Mg (II), and **Pb (II)**
*Potato-dextrose agar* (Gupta et al., 2016) (plant source)	9.0	Microwave	0.11	0–20	Cr (III), Cu (II), **Pb (II)**, Cd (II), Mg (II), Hg (II), and Ni (II)
*Lantana camara berries and EDA* (Bandi et al., 2018) (plant source)	33.1	Hydrothermal	9.64	0–200 × 10^−3^	Na (I), K (I), Mn (II), Ba (II), Fe (II), Cu (II), Sn (II), Cr (III), Al (III), **Pb (II)**, Ni (II), Mg (II), Zn (II), Hg (II), Cd (II), Ca (II), and Fe (III)
*BSA* (Wee et al., 2013) (animal source)	–	Acid hydrolysis	5.05 ×10^3^	0–6 ×10^−3^	Ag (I), Cu (II), Co (II), Hg (I), Ni (II), Mg (II), Ca (II), and **Pb (II)**
*Sago waste* (Tan et al., 2014) (plant source)	–	Pyrolysis	7.49 ×10^3^	0.2–0.8	Cr (II), Co (II), Ni (II), Al (III), Ca (II), Zn (II), Sn (II), Hg (II), **Cu (II)** and **Pb (II)**
*Bamboo* *leaves* (Liu et al., 2019d) (plant source)	–	Solvothermal	0.14	0.6–800 × 10^−3^	Al (III), Fe (III), Cr (III), Cu (II), Mg (II), Zn (II), Ca (II), Cd (II), Mn (II), Co (II), Ag (I), Na (I), K (I), **Pb (II),** and **Hg (II)**
*Biomass* (Jing et al., 2019) (Biomass source)	22.6	Hydrothermal	–	1.3–106.7	Ni (II), Fe (III), Fe (II), **Pb (II)**, Co (II), Zn (II), Cr (III), Cu (II), and Mn (II)
*Table sugar* (Ansi and Renuka, 2018) (plant source)	2.5	Microwave	67	–	Cd (II), Hg (II), Cu (II), Fe (III), K (I), Na (I), Ni (II), Co (II), Cr (VI), Mn (II), Ca (II), Zn (II), and **Pb (II)**
*Chocolate (2016)* (Liu et al., 2016)	–	Hydrothermal	12.7	0.033–1.67	**Pb (II),** Hg (II), Fe (III), Cu (II), As (III), As (V), Mn (II), Zn (II), Al (III), Mg (II), Ni (II), Cd (II), Co (II), Ba (II), Ca (II), Sn (II), Fe (II), Ag (I), Na (I), and K (I)

Commonly used salts for Pb(II) detection : Pb(NO_3_)_2_, PbCl_2_, Pb(CH_3_COO)_2_ (Selectivity marked in bold letters; last column).

*Ocimum sanctum* leaves (Bhatt et al., 2018) derived CDs revealed high stability in aqueous solution and were highly specific and selective toward the Pb (II) ions. The proposed nanoprobes were used to detect Pb (II) in real water samples and triple-negative breast cancer cells (MDA-MB 468 cells). The Pb (II) ions effectively quench the FL signal by electron-hole recombination process because of high binding affinity between vacant d-orbital of Pb (II) and -NH_2_ group on the surface of CDs. This consequence is spotted majorly because Pb (II) is a heavy metal that tends to grab an electron pair donated by the nitrogen atom of the amine group.

A similar process occurs closer to the surface of CDs, which facilitates quenching in the FL signal intensity. Shui Wee et al. (Wee et al., 2013) reported the first study on Pb (II) sensing by the electron transfer mechanism using CDs prepared by protein-based BSA. Although a similar quenching mechanism was observed in CDs synthesized from Sago waste (Tan et al., 2014) from industry and chocolate (Liu et al., 2016), Xing Xu et al. reported that the CDs made from flavonoid extracts of *Ginkgo biloba* leaves (Xu et al., 2018) reveal flavonoid moiety on the edges of the CDs during the fabrication process to bind Pb (II) selectively. In addition, such CDs doped with agarose hydrogel improve visual detection and removal of Pb (II) both in buffer and environmental water samples.

Rajkumar Bandi et al. (Bandi et al., 2018) prepared NCDs from Lantana Camara berries and reported an in-depth understanding of the FL quenching mechanism using static and dynamic/collision processes. In this study, the static/dynamic quenching was studied using Stern-Volmer plots obtained at three different temperatures revealing that the quenching constant is directly proportional to temperature. This was further confirmed through lifetime and absorbance studies in Pb (II) in CDs aqueous solution. These NCDs successfully detected Pb (II) in the real water, human sera (serum and urine) samples and under intracellular conditions. Further, V. A. Ansi et al. (Ansi and Renuka, 2018) prepared CDs (spherical nanoparticles of size 3.5 nM) using table sugar. They reported carboxylate groups' involvement in the aggregation process using FTIR and XRD results. Reliability of the system is confirmed by analyzing real water samples with a turbidimeter and successfully detecting Pb (II) in real water samples and triple-negative breast cancer cells (MDA-MB 468 cells). No interference studies were reported with a mixture of metal ions. The potato-dextrose agar (PDA) , was used as a precursor to form CDs for successful detection in solutions and paper-based sensor strips. FL quenching might be because of an excited state electron transfer reaction.

Further, the complex formation of CDs and Pb (II) was confirmed by the decrease in zeta potential (Gupta et al., 2016). Guanhong Liu et al. (Z. Liu et al., 2019d) prepared multi emission fluorescent nanohybrids CDs using extracts from bamboo leaves for sensing Pb (II) in real river water and projected their usage in waste management, water safety, and environmental monitoring. The biomass from Hemicellulose, Cellulose, and chitosan were used to make CDs (uniform size: 2–4 nm) with high sensitivity and excellent quantum yield. Jing et al. changed the surface state of CQDs by modifying the oxidation conditions, leading to improved FL efficiency (Jing et al., 2019). The Pb (II) detection limit was reported to be much lower than the permitted value recommended by WHO in drinking water and exhibited good photostability with possible usage as a sensing probe for Pb (II) in real samples.

### As (III)

Out of oxidation states As(III) and As(V) of Arsenic, As(III) is more toxic than As(V) (Del Razo et al., 1990). The poisonous action of As (III) adversely affects the environment, water quality, causing human health hazards like cardiovascular, respiratory diseases, and various types of cancer. The WHO declared it as a global environmental problem for its higher concentration than 10 ppb in natural water. The U.S. EPA and International Association for Cancer Research certified it as a Category one and a Group A human carcinogen (Yogarajah and Tsai, 2015; Chauhan et al., 2017; Zhou et al., 2018). Chemosensors, biosensors, iodometric, and polarographic methods are all standard analytical methods for accurately detecting As (III) ions. Even though these approaches are extremely qualitative and quantitative, they are limited by instrumentation cost, cumbersome analytical methodologies, and time-consuming material preparation methods (Radhakrishnan and Panneerselvam, 2018). Green synthesized carbon dots have evolved as an effective fluorescent probe with striking features for detecting As (III) ions.

Although some groups have reported As (III) sensing and reduction through citric acid derived CDs, not many groups have reported As (III) sensing through green CDs, Zahra Ramezani et al. (Ramezani et al., 2018) reported As (III) detection at pH five using Quince fruit mediated CDs (size 4.85 ± 0.07 nm) and also used cations Fe (III) and anions MnO_4_^−1^ to study their effect on PL quenching. In this work, the addition of As (III) to MnO_4_^−1^, resulted in the formation of Mn (II) and subsequent addition to CDs demonstrated an enhancement in the PL signal intensity through the electron-hole pair recombination process. Similarly, K. Radhakrishnan et al. (Radhakrishnan and Panneerselvam, 2018) studied the sensing activity of Glutathione passivated prickly pear cactus fruit-based CDs toward the detection of As (III). It was satisfactorily tested in different real water samples. The PL quenching mechanism was identified as static, using lifetime measurements. The selectivity toward As (III) and ClO^**−**^ was reported during interference study performed in the presence of other metal ions.

### Co (II)

Although Cobalt exhibits two oxidation states Co(II) and Co(III), Co(II) is more commonly available in the environment (Leyssens et al., 2017). Despite Cobalt being essential for the human body, its excessive intake can lead to many health hazards like asthma, diarrhea, low blood pressure or even death (Ahmadpour et al., 2009; Li et al., 2015a; ). In addition, its usage in many industries like mining, pigments, paints, etc. has been causing environmental pollution (Manohar et al., 2006; Rafighi et al., 2010). The safe limits of watering for irrigation and livestock are 0.05 and 1.0 mg/L as per guidelines laid by the Environmental Bureau of Investigation and Canadian Water Quality (Awual et al., 2014). For detection of Co (II), there are many techniques like ICP-OES (Inductively Coupled Plasma- Optical Emission Spectroscopy), atomic absorption spectrometry, chemiluminescence, etc (Dutta et al., 2020). In comparison to these conventional methods, the fluorescence-based technique has some interesting features due to its economic viability. CDs based on natural precursors have emerged because of their excellent fluorescence characteristics.

N-doped CDs (1–10 nM) were prepared using Nerium Oleander L. Petals (as carbon source) and Ethylene Diamine (Nitrogen source) (Dutta et al., 2020). On the addition of Co (II), the emission intensity of NCQDs and Rhodamine 6G (Rh6G) system is quenched simultaneously, probably because of the FRET process getting hindered and the metal ions getting adsorbed through the Brownian movement (Dutta et al., 2020). Recently, Chunxi Zhao et al. (Zhao et al., 2019a) reported nitrogen-doped CDs using Kelp and ethylenediamine for the visual detection of Co (II) and tested in real water samples. The possible FL quenching mechanism was identified as IFE through overlapping of absorbance/FL signals and unvarying lifetime measurements.

### Al (III)

Aluminum is one of the most abundant elements in Earth’s crust. It reacts with various biomolecules resulting in health hazards like Alzheimer's disease, Parkinson's disease, softening of bones, and breast cancer (Martyn et al., 1989; Burwen et al., 1995; Flaten, 2001; Darbre, 2005). The safe limit of Al (III), set up by WHO, is ∼3–10 mg per day and 7 mg/kg per week (based on body weight) (Barceló and Poschenrieder, 2002). Various analytical methods like inductively coupled plasma mass spectrometry (ICP-MS), atomic emission/absorption spectrometry, voltammetry, high-performance liquid chromatography, and recently gold and silver nanoparticles-based colorimetric methods are used for the measurement of Al (III) ion in numerous samples. Besides these methods, FL CDs generated from green precursors are gaining attention.

Jigna R. B. et al. (Bhamore et al., 2018) found “turn-on” chelation enhanced FL (CHEF) mechanism for Al (III) detection using hydrothermally fabricated pear fruit based CDs. The CHEF mechanism was explained using hard acid and donor (i.e., carboxylic and amine) groups' interactions to form Al (III) ion-CDs complexes. Although the developed nanosensor was also tested in real water samples, no interference in the presence of cations, anions, and pesticides was reported.

### Ag(I)

Three oxidation states of Silver Ag(I), Ag(II), and Ag(III) exist (McMillan, 1962). Ag (I) is one universal contaminant having hazardous effects on the aquatic environment and human health (World Health Organization, 1996). The U.S. EPA has set up the permissible limit for Secondary Maximum Contaminant Level (SMCL) for Ag as 0.1 mg/L (World Health Organization, 1996; Technical application bulletin, 2004). Analytical procedures ICP-AES and ICP-MS are used to detect Ag nanoparticles (Akhgari et al., 2020). These techniques are not able to distinguish nanoparticles from ions and require costly instruments. To address these issues, FL sensors using green-derived CDs are currently being developed.

Nandhini et al. (Arumugam and Kim, 2018) prepared water-soluble CQDs (ws CQDs) using Broccoli juice for the selective detection of Ag (I). Photoluminescence quenching is because of energy transfer between the Ag (I) and oxygen functional groups on the surface of the CQDs. On the other hand, the amine-terminated Graphene Quantum dots (Am-GQDs) prepared using pyrolysis of waste biomass (dead Neem leaves) have shown FL quenching in the presence of Ag (I) and the corresponding regeneration (switch on) upon the addition of L-cysteine (Suryawanshi et al., 2014). In addition, the N-CDs synthesized using Pomegranate Juice (carbon source) and Ammonium Hydroxide (nitrogen source) (particle size 2–5 nM) have been used for the detection of Ag (I). NCDs and Ag nanoparticles got aggregated in the presence of L-cysteine, resulting in the FL quenching of NCDs. These CDs revealed no interference in the presence of other metal ions and have been satisfactorily used in the analysis of spiked river water samples to detect Ag (I) (Akhgari et al., 2020).

### Au (III)

Gold exists in two oxidation states Au (I) and Au (III) (Bergendahl, 1975). Au (III) is potentially more toxic to the human body as compared to metallic gold. The large quantity of gold-containing waste released to the environment produces hazardous effects on human health and leads to adverse damage to the liver, kidney, and peripheral nervous system and affects the ecosystem by inhibiting plants' growth. Therefore, it is necessary and essential to detect Au (III) ions both in the living systems and environment (Adler et al., 2007; Ramanan et al., 2018). AFM (Atomic Fluorescence Microscopy), AAS (Atomic absorption Spectroscopy), and ICP-MS are the analytical techniques that are conventionally used for the detection of Au (III) (Liao et al., 2016; Raji et al., 2019). Although developing highly sensitive and effective fluorescent probes is still a challenge, the utilization of green CDs to detect Au (III) has been reported.

Jie Liao et al. (Liao et al., 2016) presented Au (III) detection in deionized and river water using N-CDs from natural peach gum polysaccharide and ethylenediamine. Rahmani et al. (Rahmani and Ghaemy, 2019) prepared environment and photostable NCDs using gum tragacanth (GT) and ethylenediamine. The FL quenching mechanism was explained on the basis of both synergetic effect (electron transfer from Au (III) to CDs) and FRET (enhancement in the absorption signal). Interference results confirmed the specific selectivity with high sensitivity for Au(III). Recently, Raji et al. (Raji et al., 2019) reported the reduction of Au (III) to polydisperse Au nanoparticles during the detection of Au (III) from Jackfruit seeds derived N-CDs.

The main works describing As (III), Co (II), Al (III), Au (III), and Ag (I) sensing through green precursor generated CDs have been tabulated (Table 7) below.

**Table 7:** Detection of Arsenic (III), Cobalt (II), Aluminum (III), Gold (III), and Silver (I) using green precursors derived CDs

Precursor (Year)	Quantum yield (%)	Techniques used	Limit of detection, μM	Linear concentration range, μM	Metal ions screened for selectivity
**Arsenic (As (III))**					
*Edible prickly pear cactus* (Radhakrishnan and Panneerselvam, 2018) (plant source)	12.7	Hydrothermal	2.3 × 10^−3^^a^	2–12 × 10^−3^^a^	**As (III)**, Ag (I), K (I), Ca (II), Cu (II), Ni (II), Ba (II), Pb (II), Hg (II), Cd (II), Co (II), Fe (III), and Fe (II)
*Quince fruit* (Ramezani et al., 2018) (plant source)	8.55	Microwave	0.01	0.5–10	**As (III)**, Fe (III), Ag (I), Mg (II), Co (II), Zn (II), Cu (II), Al (III), Mn (II), Ni (II), Hg (II), Cr (III), Cd (II), and Fe (II)
**Cobalt (Co (II))**					
*Kelp (2019)* (Zhao et al., 2019a) (algal source)	23.5	Microwave	0.39	1–200	K (I), Mn (II), Cd (II), Fe (II), Ni (II), Cu (II), Na (I), Hg (II), Pb (II), Fe (III), Cr (III), Cr (VI), and **Co (II)**
*Nerium Oleander L*. *Petals + EDA* (Dutta et al., 2020) (plant source)	3.5	Hydrothermal	6.45 × 10^−3^	0–40	Na (I), K (I), Ca (II), Cd (II), Ag (I), Mg (II), Cr (III), Mn (II), Fe (II), Fe (III), Ni (II), Cu (II), Zn (II), **Co (II),** and Hg (II)
**Aluminum (Al (III))**					
*Pyrus pyrifolia (Pear) Fruit* (Bhamore et al., 2018) (plant source)	10.80	Hydrothermal	2.5 × 10^−3^	0.005–50	Hg (II), Ni (II), Ca (II), K (I), Ba (II), Cd (II), Cr (III), Cu (II), Pb (II), Zn (II), Fe (III), and **Al (III)**
**Silver (Ag (I))**					
*Broccoli* (Arumugam and Kim, 2018) (plant source)	–	Hydrothermal	0.5	0–600	Cr (III), Mn (II), Ni (II), **Ag (I)**, Cd (II), Cu (II), Ca (II), Sn (II), Zn (II), Co (II), and Fe (III)
*Neem leaves* (Suryawanshi et al., 2014) (plant source)	54	Pyrolysis and Hydrothermal	0.2–0.6 ×10^3^^a^	–	Cu (II), Ni (II), Co (II), Fe (II), Fe (III), **Ag (I)**, Hg (II), and Pb (II)
*Pomegranate Juice + Ammonium Hydroxide* (Akhgari et al., 2020) (plant source)	–	Hydrothermal	38 × 10^−3^	8.3 × 10^−4^ 3.3 × 10^−2^	**Ag (I)**, Al (III), Fe (II), Na (I), Ni (II), Mg (II), Zn (II), and Cu (II)
**Gold (Au (III))**					
*Jackfruit seeds + o-Phosphoric acid* (Raji et al., 2019) (plant source)	17.91	Microwave	239 × 10^−3^	0–100	Na (I), K (I), Ca (II), Mn (II), Fe (II), Fe (III), Co (II), Cu (II), Zn (II), Ag (I), Hg (II), Pb (II), and **Au (III)**
*Peach gum Polysaccharides + Ethylenediamine (2016)* (Liao et al., 2016) (plant source)	28.46	Hydrothermal carbonization	0.64	0–50	Na (I), Ag (I), Zn (II), Ca (II), Mn (II), Ni (II), Cd (II), Cu (II), Co (II), Pb (II), Fe (II), Fe (III), Pd (II), Pt (IV), and **Au (III)**
*Gum tragacanth (GT) + EDA* (Rahmani and Ghaemy, 2019) (plant source)	66.74	Hydrothermal	2.69	0–100	Na (I), **Au (III)**, K (I), Mg (II), Ca (II), Cr (III), Sn (II), Ba (II), Mn (II), Fe (II), Co (II), Ni (II), Cu (II), Zn (II), Ag (I), Cd (II), Hg (II), Fe (III), Ti (III), Al (III), and Pb (II)

Commonly used salts for Cobalt (II), Aluminum (III), Gold (III), and Silver (I) detection were CoCl_2_, AlCl_3_, AuCl_3_ and AgNO_3_ respectively. (Selectivity marked in bold letters; last column).

a Denoted values were recalculated for uniformity in the corresponding units with respect to other reports.

**Figure 6:**
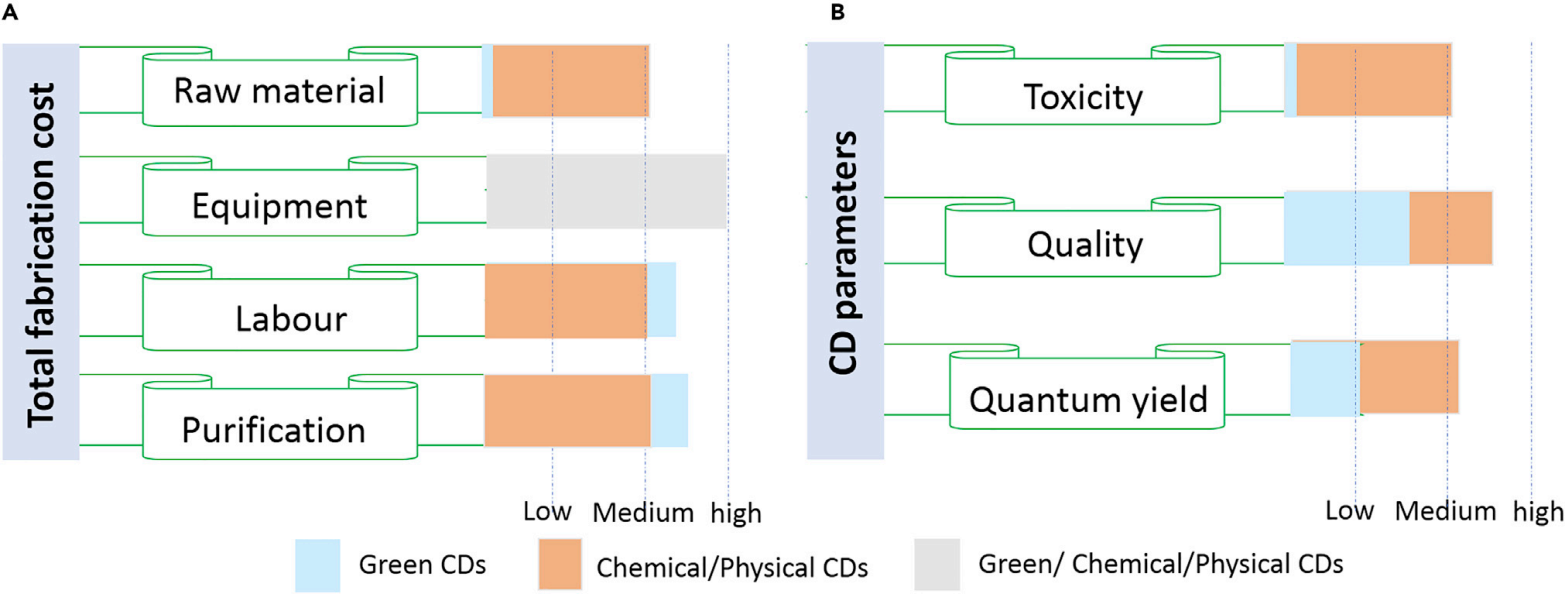
Green vs chemical/physical synthesis of CDs Comparison (green vs chemical/physical technique for CDs' preparation) chart represents the (A) qualitative estimation of total fabrication cost range and (B) parameters to be considered during CDs preparation process.

Chemical substances were usually used as carbon precursors in many studies. In contrast, green natural substances have become increasingly popular as carbon sources in recent years because of their environmental friendly and readily available features. In addition, we present an idea of an overall fabrication cost and parameters to be considered for the fabrication of CDs, in the qualitative comparison charts (Figure 6).

## Research challenges and future perspectives

This overview on the plant-mediated green carbon dots and their recent progress in the optical detection of major environmental contaminants (heavy metal ions) reveals their escalated development in recent years. However, some challenges still need to be addressed for their possible scalability and application as economically viable daily life sensing probes. Apart from the synthesis strategies for developing highly stable and efficient green CDs, the emission from the entire visible spectrum and narrow bandwidth of fluorescence signal is required for specific applications and enhanced sensitivity. The FL signal intensity and quantum yield of green CDs are still low compared to traditionally prepared chemically synthesized (carbon-based or CdTe, CdSe, CdS, etc.) quantum dots. Notably, researchers are still struggling to find the possible economically viable techniques/methods for purifying the green CDs.

Several less explored sustainable precursors including recycled waste, biomaterials, and residuals, need to be evaluated for the fabrication of naturally doped CDs with high quantum yield. Besides the required mechanistic understanding of the formation of green CDs, it is indispensable to identify the origin behind the precursor-based specificity of the CDs toward specific metal ions. On the other hand, a simultaneous and straightforward surface modification could increase optical signal for enhanced applicability. More exhaustive studies are required to develop a ratiometric and reusable (by functionalizing CDs onto 2D/3D substrates) sensing probes having FL emission in the UV-visible-NIR region. We believe that the upcoming exploration of sensitive optical detection systems using carbon dots will gain extensive attention in food, agriculture, and textile pollutant sensing because of its simplicity, biocompatibility, and cost-effectiveness.

### Possible strategies to improve overall fabrication efficiency from biomass

Plant-based biomass materials are receiving increased attention as an abundant, renewable, and economical alternative to chemicals to produce numerous value-added products. Industrial production of a wide range of value-added sustainable materials depends on biowastes, including energy crops, agricultural biomass residues, forest biomass, and food-based biomass wastes. The efficiency of fabrication technologies depends on the types of biomass used as raw materials that differ in the contents and compositions of carbon and their functional groups. Compared to other chemical fabrication technologies, apart from cleaning and drying biomass materials must be broken into smaller components, where hydrolysis and pretreatment (e.g., grinding and extract preparation) can play a vital role in the overall efficiency of the carbon dots. In addition, the presence of diversity in the chemical composition of biomass can also improve efficiency in the production of carbon dots. Apart from the heteroatom-rich (nitrogen, phosphorus, and sulfur) biomass, the use of green precursors with some specific metal ions can enhance the quantum efficiency of the CDs.

### Possible steps to improve sensitivity and selectivity of individual metal ions

One of the major disadvantages in green CDs is their possible selectivity toward the multiple metal ions detection. Appropriate simple and easy to use functionalization protocols, without losing the optical, chemical, and biological properties of the nanomaterials, are necessary to get the improved selectivity and sensitivity of CDs toward heavy metal ions. Apart from that, using the sustainable genetically engineered biomass with specific metabolic pathways can help in the development of finger-printed optical sensing response patterns. Similar to selectivity, signal amplification is the direct method for improving the sensitivity of optical sensors. One of the easy and prominent ways to improve the signal intensity is by passivating the CDs with different polymer-based functionalization protocols. Hence, a requirement-based balance between the selectivity and sensitivity protocols can be achieved. In addition, combination of metal/semiconductor nanoparticles with CDs (here, nanoparticles can be used as carriers to load many active species) can possibly result in desirable optical properties. These nanomaterials can facilitate achieving an enhanced signal amplification and act as an ultrasensitive optical sensor to detect analytes (Khanal et al., 2012).

Finally, with the increasing demand for home testing and personalized healthcare, a vast number of studies on detection systems are needed. There is a trend for miniaturization and facilitation of detection using portable and small-sized devices that provide rapid and accurate responses with potential importance in the point-of-care technologies. Hardware and software can be combined with detection protocols such as colorimetric testing and luminescent assays using mobile, hand-held (lateral flow test strips, microfluidic strips), and wearable devices to function adequately.
